# Effects of fractional inspired O_2_ on the O_2_ pathway during submaximal and maximal exercise in male endurance athletes

**DOI:** 10.14814/phy2.70639

**Published:** 2025-10-29

**Authors:** Elias Lehtonen, Dominique D. Gagnon, Antti‐Pekka E. Rissanen, Tom Mikkola, Juha E. Peltonen

**Affiliations:** ^1^ Helsinki Sports and Exercise Medicine Clinic (HULA), Foundation for Sports and Exercise Medicine Helsinki Finland; ^2^ Sports and Exercise Medicine, Faculty of Medicine University of Helsinki Helsinki Finland; ^3^ Faculty of Sport and Health Sciences University of Jyväskylä Jyväskylä Finland; ^4^ School of Kinesiology and Health Sciences Laurentian University Sudbury Canada; ^5^ School of Electrical Engineering Aalto University Espoo Finland

**Keywords:** convection, diffusion, hyperoxia, hypoxia, oxygen transport

## Abstract

We utilized non‐invasive methods and novel computational approaches to examine the effects of acutely varying fractional inspired O_2_ (FIO_2_) on convective and diffusive steps of O_2_ transport and muscle tissue (de)oxygenation during incremental cycling to exhaustion in 10 Tier 3 and 4 endurance athletes breathing either 0.152, 0.209, or 0.298 FIO_2_. At submaximal work rates (100–275 W) in hypoxia, higher cardiac output compensated for lower arterial O_2_ content. At maximal work rate, convective O_2_ transport was lower in hypoxia (mean [95%CI]: 5.37 [5.14–5.59] L/min, *q* < 0.0001) and higher in hyperoxia (6.84 [6.50–7.18] L/min, *q* = 0.043) compared to normoxia (6.56 [6.16–6.95] L/min), whereas O_2_ diffusive conductance did not differ between conditions (94 [82–106], 98 [83–112], 98[87–109] mL/min/mmHg for hypoxia, normoxia and hyperoxia, respectively, *p* = 0.490). Consequently, maximal O_2_ uptake (V̇O_2max_) was lower in hypoxia (4.06 [3.85–4.27] L/min, *q* < 0.0001) and higher in hyperoxia (5.02 [4.85–5.19] L/min, *q* = 0.003) compared to normoxia (4.83 [4.63–5.02] L/min). In hypoxia, muscle tissue saturation index was 1%–4%‐units lower compared to normoxia and hyperoxia during submaximal cycling but similar at maximal work rate. In summary, central and peripheral compensatory mechanisms maintained O_2_ uptake despite altered FIO_2_ at submaximal work rates. At maximal work rate the effects of hypoxia and hyperoxia on V̇O_2max_ were mediated through convective O_2_ transport.

## 1 INTRODUCTION

Maximal O_2_ uptake (V̇O_2max_) is one of the key determinants of endurance performance and a vital sign of health and well‐being (Millet et al., 2023). The determinants of V̇O_2max_ are best understood by considering the O_2_ pathway from inspired air to the mitochondria. O_2_ transport consists of four elemental steps, alternating convective and diffusive mechanisms: (1) ventilation, (2) alveolar‐capillary diffusion, (3) circulation, (4) diffusion from capillaries to mitochondria (Wagner, 2023). The integrated nature of the steps of O_2_ transport can be understood by conflating the Fick principle with Fick's law of diffusion and graphically expressed as the “Wagner diagram” (Wagner, 2011). Every step of the O_2_ cascade together defines the end outcome, expressed as V̇O_2max_ (Wagner, 2011). This integrated model represents the current gold standard for understanding the determinants of V̇O_2max_ (Porcelli et al., 2023; Roca et al., 1989; Wagner, 1991, 1992).

Due to the invasive nature of the measurements typically used for solving the variables needed for plotting the Wagner diagram, the approach remains underutilized, particularly at the level of whole‐body V̇O_2max_ (Millet et al., 2023). Consequently, the contributions of central and peripheral components of V̇O_2max_ are frequently misinterpreted particularly so that arterial‐mixed venous O_2_ content difference (C(a‐⊽O_2_)) is frequently but erroneously used as an indicator of the peripheral component of V̇O_2max_ (Gifford et al., 2024). Therefore, the relative contributions of central and peripheral determinants to V̇O_2max_ are misunderstood (Goulding, 2024; Poole & Musch, 2008). However, as proposed by Wagner (Wagner, 2023), a reasonable non‐invasive compromise can be achieved by measuring whole‐body V̇O_2_ at the mouth, coupled with non‐invasive measures of cardiac output (Q̇) and an estimate of arterial O_2_ content (CaO_2_).

Manipulations of partial inspired O_2_ pressure have been used to study the effects of altered O_2_ availability as well as to uncover the physiological mechanisms of O_2_ transport and utilization. At submaximal intensities O_2_ uptake (V̇O_2_) is maintained in acute moderate hypoxia, but V̇O_2max_ is reduced due to compromised maximal convective O_2_ transport (Q̇aO_2max_) (Calbet et al., 2003; Ferretti et al., 1997; Hartley et al., 1973; Hughes et al., 1968; Knight et al., 1993; Stenberg et al., 1966; Wehrlin & Hallén, 2006). Previously, hyperoxia has been shown to elevate leg or skeletal muscle V̇O_2max_ by 8%–18% in trained (Knight et al., 1993; Richardson, Leigh, et al., 1999) but not in untrained subjects (Cardús et al., 1998). Leg V̇O_2max_ decreases linearly with fractional inspired O_2_ (FIO_2_) when plotted against partial O_2_ pressure in mean capillary or femoral venous blood in trained but not in untrained individuals, and thus, manipulating FIO_2_ reveals differing limitations to V̇O_2max_ depending on training status (Broxterman et al., 2024; Gifford et al., 2016; Roca et al., 1989; Roca et al., 1992). However, the past studies, which have been conducted in hypoxia, normoxia and hyperoxia and conflated the Fick principle and Fick's law of diffusion, have been constrained to either small muscle mass exercise (Broxterman et al., 2024; Pedersen et al., 1999; Richardson, Leigh, et al., 1999) or have only measured leg V̇O_2max_ during large muscle mass exercise (Cardús et al., 1998; Knight et al., 1993). During single‐leg knee extension exercise, leg blood flow increases are 120% higher compared to cycling, whereas muscle specific maximal diffusive O_2_ conductance (ḊO_2max_) is 50% lower due to lower mitochondrial excess capacity and consequently lower mitochondrial O_2_ affinity (Cardinale et al., 2019). Furthermore, during whole‐body exercise, partial O_2_ pressure of mixed venous blood (P⊽O_2_) is consistently 30%–40% higher compared to femoral venous blood leading to potentially significant variations in local and whole‐body determinants of O_2_ transport (Calbet et al., 2005). In the context of athletic performance, the focus is most often on whole‐body V̇O_2max_. Thus, we aim to extend the application of the gold standard theoretical framework to this context. To our knowledge, whole‐body V̇O_2max_ limitations have not been previously assessed with the Wagner model in trained subjects with both hyperoxia and hypoxia compared to normoxia.

Muscle oxygenation responses, monitored with near‐infrared spectroscopy (NIRS), provide insights into the local balance between O_2_ transport and utilization (Barstow, 2019). While NIRS‐derived variables have been suggested to represent the terms of the Fick principle and Fick's law of diffusion (Barstow, 2019; Boone, Vandekerckhove, et al., 2016; Goulding et al., 2021; Grassi & Quaresima, 2016; Manferdelli et al., 2023; Murias et al., 2013), it remains strongly contested whether NIRS can be utilized to solve the Fick Equations (4). Therefore, the relationship between NIRS variables, which are measured locally but influenced by all systemic responses of the O_2_ pathway, and whole‐body O_2_ transport remains incompletely understood. By assessing convective and diffusive determinants of whole‐body V̇O_2max_ in conjunction with peripheral muscle (de)oxygenation we aim to examine the interplay of local and whole‐body O_2_ transport and utilization in low and high FIO_2_.

The aim of this study was to non‐invasively and with novel computational approaches (Rissanen et al., 2024) assess central and peripheral determinants of O_2_ transport in endurance athletes during incremental cycling in normoxia, acute moderate hypoxia and hyperoxia. By focusing on both submaximal and maximal cycling in low and high FIO_2_ we sought to study limitations to and compensatory mechanisms of O_2_ transport and utilization during whole‐body exercise. Finally, by utilizing non‐invasive methods, we aimed to widen the application of the current gold standard theoretical framework for interpreting the O_2_ pathway and its limitations to V̇O_2max_.

## 2 MATERIALS AND METHODS

### 2.1 Ethical approval

The study was approved by the Local Ethics Committee of the Hospital District of Helsinki and Uusimaa (HUS/41/2023) and conducted in compliance with the Declaration of Helsinki, except for registration in a database. Participants provided written informed consent before experimental testing.

### 2.2 Study participants

Ten Tier 3 (Highly Trained/National Level) or Tier 4 (Elite/International Level) Caucasian male cyclists and speed skaters (age = 27.9 ± 8.6 years, V̇O_2max_ = 65.4 ± 4.7 mL/kg/min, height = 180.9 ± 5.1 cm, body mass = 74.0 ± 4.9 kg) volunteered to participate and gave written informed consent. Cyclists were recruited from local clubs around Helsinki, Finland and the speed skaters from the Finnish national team. The cyclists were required to compete at the highest national level (Elite‐category) and the speed skaters to specialize in the distance events (1000–10,000 m). Additionally, the participants were required to have no preceding exposure to hypoxic environments up to 3 months prior to the study measurements.

### 2.3 Study design

The experimental protocol consisted of pre‐exercise measurements and a cardiopulmonary exercise test (CPET). The subjects were advised to avoid eating and caffeine 2–3 h before their visit, abstain from alcohol for 24 h, and avoid strenuous training 1–2 days prior to their visit. On their first visit, the participants completed a survey concerning their personal health and medical history. The pre‐exercise measurements at rest included blood pressure and a 12‐lead ECG. A physician examined the participant to ensure suitability for exercise testing. Body composition was estimated by the bioimpedance method (InBody 720, Biospace Co., Ltd., Seoul, South Korea) on each visit.

The participants visited the laboratory (altitude 40 m above sea level) three times within 1 month. On each of their visits, they performed a CPET under differing conditions of FIO_2_: normoxia (FIO_2_ 0.209), hypoxia (FIO_2_ 0.152, equal to ~2900 m), and hyperoxia (FIO_2_ 0.298) in a randomized order. The hypoxic FIO_2_ was chosen to match altitudes typically experienced by endurance athletes in training and competition. The hyperoxic FIO_2_ was chosen because it should sufficiently elevate CaO_2_ compared to normoxia, but is low enough to permit the use of the Haldane‐transformation (Eschenbacher, 2017). The hypoxic and hyperoxic gases were supplied by Woikoski Medical (Woikoski Oy, Järvenpää, Finland) and were balanced with nitrogen. During the normoxic test, the participants breathed regular room air. The participants were blinded to the gas mixture. During the tests, a two‐way non‐rebreathing valve (Hans Rudolph, Kansas City, USA) was attached to the flow sensor. The valve was attached to a 150 L Douglas Bag with a hose and the Douglas Bag was continuously filled with either hypoxic or hyperoxic gas mixture unknown to the participant. In the normoxic condition, unknown to the participant, the hose was connected to room air instead of the Douglas bag.

### 2.4 Exercise test protocol

All CPETs were performed between 9 a.m. and 4 p.m. under similar environmental conditions (altitude 40 m above sea level, barometric pressure 761 ± 7 mmHg, temperature 21 ± 1°C, relative humidity 53 ± 15%) with airflow of 2–3 m/s towards the participants' frontal surface. Each individual performed all their CPETs during the same time of day. Strong verbal encouragement was given during the tests to ensure maximal effort. All testing was performed on the participants' own bicycles mounted onto an electrically braked cycle ergometer (Kickr V6, Wahoo Fitness LLC, Atlanta, GA, USA). Prior to the test, the front derailleur was shifted to the “small” chain ring, and the rear derailleur to the middle of the cassette to ensure a straight chain‐line. The participant was asked to use the same gear combination for all three CPETs.

The CPET started with 5 min of baseline unloaded cycling in normoxia after which the FIO_2_ mixture was switched to the given experimental condition, and unloaded cycling was continued for an additional 5 min. During unloaded cycling the resistance of the cycle ergometer was set at 0 W, and the participants were asked to maintain a steady cadence of 60 rpm. Subsequently, the step protocol was started at 100 W, followed by 35 W increases every 3 min. The participants were advised to maintain an optimal self‐selected cadence during the test. The participants continued exercising until task failure, defined as an inability to maintain a cadence of 60 rpm or above.

### 2.5 Cardiopulmonary responses

Gas exchange was continuously monitored breath by breath during the CPET with a computerized metabolic system (Vyntus CPX, Vyaire Medical, Mettawa, IL, USA). Before each test, the system was calibrated for flow and gas concentrations according to instructions specified by the manufacturer. The data were exported in a breath‐by‐breath and 30 s average format for subsequent analyses.

First ventilatory threshold (VT1) was identified for each individual in each FIO_2_ condition by an experienced exercise physiologist using breath‐by‐breath gas exchange data by inspecting the CO_2_ removal (V̇CO_2_) versus V̇O_2_ slope (V‐slope method) and minute ventilation (V̇E) versus V̇O_2_ for distinct breakpoints (Beaver et al., 1986; Keir et al., 2022). V̇O_2_ values for VT1 were converted to work rate (W_VT1_) by assuming a linear relationship between V̇O_2_ and work rate.

Heart rate (HR) and electrical activity of the heart were continuously monitored with a 2‐lead ECG (Powerlab, ADInstruments, Oxford, UK). Stroke volume (SV) and Q̇ were monitored with impedance cardiography (ICG) (PhysioFlow PF‐07 Enduro, Manatec Biomedical, Paris, France). The device has been validated against the direct Fick method (Charloux et al., 2000; Richard et al., 2001). To optimize signal quality, the skin was prepared according to manufacturer instructions and the electrodes were securely taped on the skin. The participants sat still on their bicycle for 5 min prior to calibration of the device to ensure stable signal quality. During calibration of the ICG signal, blood pressure was measured automatically from the left brachial artery (Tango+, SunTech Medical, Morrisville, NC, USA). Data points with a signal quality below 80% were excluded from analyses. The ICG data were exported in a beat‐by‐beat format and later transformed into a second‐by‐second format. Pulse oximetry (Nonin 9700, Nonin Medical, Inc., Plymouth, MA, USA) was used to estimate arterial O_2_ saturation (SpO_2_) from the right earlobe during submaximal and maximal exercise.

### 2.6 Muscle (de)oxygenation

Local oxygenation was monitored from the right vastus lateralis muscle using a continuous wave NIRS system (Portamon, Artinis Medical Systems, Elst, the Netherlands). The NIRS probe consisted of one receiver and three transmitters operating at wavelengths of 761 and 845 nm. The receiver to transmitter distances were 30, 35, and 40 mm. The instrument measured concentration changes of oxygenated hemo‐ and myoglobin (∆oxy[heme]), deoxygenated hemo‐ and myoglobin (∆deoxy[heme]) and total hemo‐ and myoglobin (∆total[heme]) with respect to an initial value arbitrarily set at 0 μM. Tissue saturation index (StO_2_ = (∆oxy[heme]/∆total [heme]) × 100%) was calculated from the light attenuation slope along the distance from the three emitting points as detected by the sensor of the receiving optode (Barstow, 2019).

The NIRS device was positioned on the muscle belly, parallel to the long axis of the muscle, 10 cm above the proximal border of the patella and 5 cm laterally from the mid‐line of the thigh. The positioning was confirmed by asking the participants to contract their vastus lateralis muscle and checking by visual inspection that the measured position was on the top of the muscle belly. On the first visit, the positioning of the NIRS probe was marked with a permanent pen to ensure consistent positioning across all visits. Prior to positioning the NIRS probe, the skin at the measurement site was shaved and cleaned with alcohol and a transparent Tegaderm film (3 M Company, St. Paul, MN, USA). The probe was securely attached with tape (3 M Transpore, 3 M Company, St. Paul, MN, USA), avoiding taping around the muscle to avoid any effect on local circulation. The edges of the NIRS probe were taped with dark kinesiology tape (K‐Active, Nitto Denko Corporation, Iwadeyama, Osaki, Miyagi, Japan) to avoid contamination from ambient light. Adipose tissue thickness at the site of application of the NIRS probe was measured by a skinfold caliper to ensure that the depth of light penetration sufficiently interrogated the underlying muscle (Barstow, 2019). The sampling frequency was set at 10 Hz, and the data was later transformed into second‐by‐second data as well as 30 s averages. For ∆oxy‐, ∆deoxy‐, and ∆total[heme], the deepest (40 mm) channel, which should best represent muscle oxygenation, was used for later data analyses. The initial baseline value of 0 μM for ∆oxy‐, ∆deoxy‐, and ∆total[heme] was set by averaging the signal from the last 30 s of 5 min of unloaded cycling. Work rate and peak values for ∆oxy[heme], ∆deoxy[heme], and ∆total[heme] were calculated in relation to this baseline value.

### 2.7 Blood sampling

Arterialized capillary blood gas samples (70 μL) were drawn from the participants' earlobe and fingertip immediately after task failure. The samples were immediately analyzed using a blood gas analyzer (ABL‐90 FLEX PLUS, Radiometer Medical ApS, Brønshøj, Denmark). Arterialization of capillary blood was achieved by holding a heating bag on the earlobe and in the palm of the hand. Duplicate samples were obtained from both the earlobe and fingertip. Hemoglobin concentration at maximal exercise([Hb]_max_) was determined as the mean of the obtained duplicate samples. Arterial partial O_2_ pressure (PaO_2_) at maximal exercise (PaO_2max_) and pH at maximal exercise (pH_max_) were analyzed from the first sample drawn from the earlobe immediately after task failure. Arterialized capillary blood samples drawn from the earlobe have been shown to reflect PaO_2_ and pH with adequate accuracy, particularly during exercise (Zavorsky et al., 2007).

### 2.8 Data calculation

The data from the metabolic system, ICG, pulse oximeter, and NIRS were time‐aligned. The mean values of the last 30 s at rest, during unloaded cycling, during unloaded cycling in the experimental condition of FIO_2_, and at each work rate were selected for analysis. C (a‐⊽)O_2_ was calculated for each work rate by dividing V̇O_2_ by Q̇. CaO_2_ was calculated for each work rate with Equation 1 and mixed venous O_2_ content (C⊽O_2_) was calculated with Equation 2.

(1) $${\mathrm{CaO}}_{2}=1.34\times \left[\mathrm{Hb}\right]\times {\mathrm{SpO}}_{2}$$

(2) $$C\bar{v}O_{2}={\mathrm{CaO}}_{2}-C\left(a-⊽\right)O_{2}$$

For submaximal work rates, convective O_2_ flow (Q̇aO_2_) was calculated by multiplying Q̇ with CaO_2_, and O_2_ extraction fraction was calculated by dividing C(a‐⊽)O_2_ by CaO_2_.

For work rate‐wise comparisons of respiratory, hemodynamic, and muscle oxygenation data, values from unloaded cycling, unloaded cycling in the experimental condition of FIO_2_, submaximal work rates between 100 and 275 W, and maximal work rate were used to ensure a similar number of data points for each FIO_2_ condition. The individual relationships between ∆deoxy[heme] and C⊽O_2_ were plotted using data from unloaded cycling, unloaded cycling in the experimental condition of FIO_2_, and each work rate.

Maximal values for V̇O_2max_ and Q̇ (*Q̇*_max_), were determined as the highest 30‐s moving average. For ∆oxy[heme], ∆deoxy[heme], ∆total[heme], and StO_2_, the value at maximal exercise was determined as the mean of the 30 s at the end of the test. Maximal work rate (*W*_max_) was calculated as the mean of the last 3 min of the CPET.

V̇O_2max_, *Q̇*_max_, [Hb]_max_, SpO_2_ at maximal exercise (SpO_2max_), PaO_2max_ and pH_max_ values were used to compute the convective and diffusive components of V̇O_2max_ with a previously published open access application (Helsinki O_2_ Pathway Tool, HO_2_PT) developed for numerical and graphical modeling of the Wagner diagram (Rissanen et al., 2024). Temperature was approximated at 38.5°C (González‐Alonso et al., 2015), and the constant (k) in Equation 5 was set at 2 based on previously reported values (Broxterman et al., 2024; Calbet et al., 2005; Richardson, Grassi, et al., 1999; Richardson, Leigh, et al., 1999; Roca et al., 1989, 1992). Briefly, the O_2_ transport and utilization cascade is defined by the Fick principle (Equation 3) and Fick's diffusion law (Equation 4). According to the law of mass conservation, both equations must be true at the same time.

(3) $$\dot{V}O_{2}=\dot{Q}\times \left({\mathrm{CaO}}_{2}-C\bar{v}O_{2}\right)$$

(4) $$\dot{V}O_{2}=\dot{D}O_{2}\times \left(P\bar{\mathrm{cap}}O_{2}-{\text{PmitO}}_{2}\right)$$

where ḊO_2_ = O_2_ diffusive conductance, $P\bar{\mathrm{cap}}$O_2_ = partial mean capillary O_2_ pressure, and PmitO_2_ = partial mitochondrial O_2_ pressure.

Equation 4 can be further simplified based on two assumptions: First, by assuming a linear relationship between $P\bar{\mathrm{cap}}$O_2_ and P⊽O_2_ and uniform (ḊO_2_) along the capillaries with homogenous blood flow distribution (Roca et al., 1989), and second, by assuming PmitO_2_ to be algebraically zero in endurance‐trained individuals at (near) maximal exercise (Roca et al., 1989). Thus, Fick's law of diffusion can be presented as follows (Equation 5) (Wagner, 2011):

(5) $$\dot{V}O_{2}=\dot{D}O_{2}\times k\times P\bar{v}O_{2}$$

where *k* = constant.

HO_2_PT was used to calculate the following components of the Wagner diagram at maximal exercise: CaO_2_ at maximal exercise (CaO_2max_), Q̇aO_2max_, C⊽O_2_ at maximal exercise (C⊽O_2max_), C(a‐⊽)O_2_ at maximal exercise (C(a‐⊽)O_2max_), P⊽O_2_ at maximal exercise (P⊽O_2max_) and ḊO_2max_. Details and limitations behind the calculations performed by the HO_2_PT are extensively discussed elsewhere (Rissanen et al., 2024).

### 2.9 Statistical analyses

Normality of the data was examined by the Shapiro–Wilk test. Normally distributed data were analyzed with repeated measures one‐way ANOVA with the Geisser–Greenhouse correction to compare the maximal values between normoxia, hypoxia, and hyperoxia. For non‐normally distributed data, the Friedman test was utilized. *W*_max_, W_VT1_, V̇O_2max_, *Q̇*_max_, [Hb]_max_, PaO_2max_, pH_max_, CaO_2max_, Q̇aO_2max_, C⊽O_2max_, ḊO_2max_ and P⊽O_2max_ were normally distributed. SpO_2max_ and C(a‐⊽)O_2max_ were non‐normally distributed. Multiple comparisons were corrected for by controlling the false discovery rate (*q* value) with the two‐stage step‐up method of Benjamini et al. (2006). For work rate‐wise comparisons, repeated measures two‐way ANOVA (work rate × treatment) with the Geisser–Greenhouse correction to compare the values at each work rate between normoxia, hypoxia, and hyperoxia was used for normally distributed variables (V̇O_2_, Q̇, HR, SV, Q̇aO_2_, CaO_2_, C⊽O_2_, O_2_ extraction, StO_2_, ∆total[heme], ∆deoxy[heme], and ∆oxy[heme]), and the Friedman test was utilized for non‐normally distributed data (SpO_2_ and C(a‐⊽)O_2_). Multiple comparisons were corrected for by controlling the false discovery rate (*q* value) with the two‐stage step‐up method of Benjamini, Krieger and Yekutieli. Work rate‐wise cardiopulmonary and muscle oxygenation data are presented as mean (SD). Values determined at maximal exercise are presented as mean (95% CI) for normally distributed variables and as median (IQR) for non‐normally distributed variables. Linear regression was used to examine the relationship between ∆deoxy[heme] and C⊽O_2_ for each individual in each FIO_2_ condition. The desired false discovery rate (*Q*) was set at <0.05, hence *q* values <0.05 are treated as discoveries. Statistical significance was accepted at the *p* < 0.05 level for linear regression and Spearman's rank correlation. All results were computed with Prism (v. 10.3.0, GraphPad Software, San Diego, CA, USA).

## 3 RESULTS

### 3.1 Effects of acutely varied FIO_2_ on O_2_ transport during submaximal exercise

**FIGURE 1:**
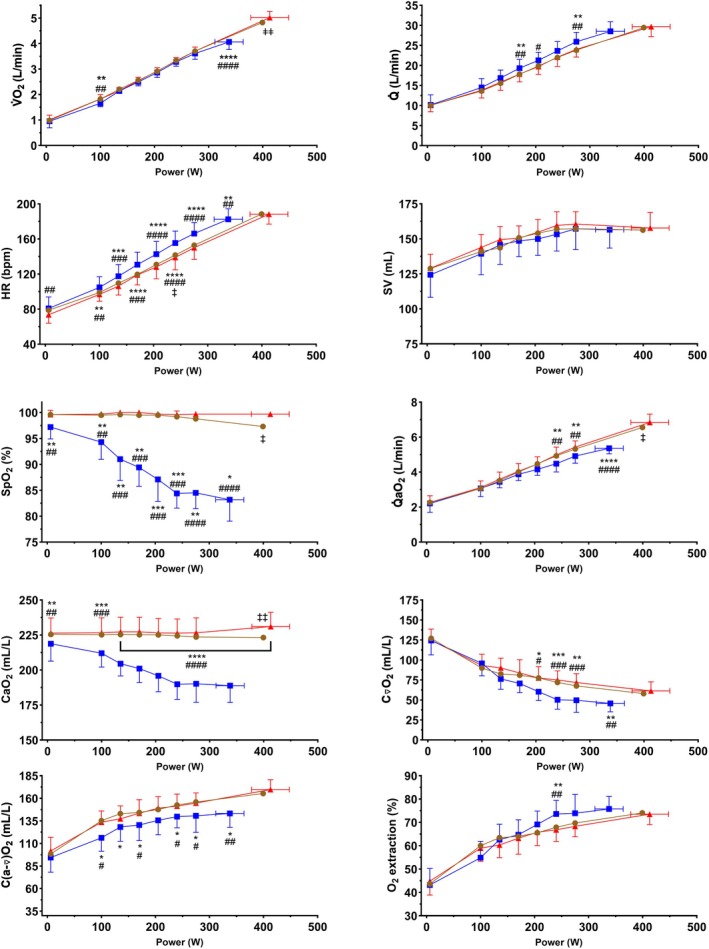
Work rate‐wise responses in determinants of O_2_ transport to exercise in normoxia (brown circles), hypoxia (blue squares) and hyperoxia (red triangles). Data are presented as mean ± SD, *n* = 10. C(a‐⊽)O_2_, arterial–venous O_2_ content difference; C⊽O_2_, mixed venous O_2_ content; CaO_2_, arterial O_2_ content; HR, heart rate; Q̇, cardiac output; Q̇aO_2_, convective O_2_ delivery; SpO_2_, arterial O_2_ saturation estimated with pulse oximetry; SV, stroke volume; V̇O_2_, O_2_ uptake. *Significant difference between hypoxia and normoxia, *q* < 0.05, **q < 0.01, ***q < 0.001, ****q < 0.0001. ^#^Significant difference between hypoxia and hyperoxia, *q* < 0.05, ^##^ *q* < 0.01, ^###^ *q* < 0.001; ^####^ *q* < 0.0001. ^‡^Significant difference between normoxia and hyperoxia, *q* < 0.05, ^‡‡^ *q* < 0.01.

Figure 1 presents work rate‐wise responses in determinants of O_2_ transport in normoxia, acute moderate hypoxia and hyperoxia. We observed main effects of work rate (*F*_2.496,22.46_ = 1237, *p* < 0.0001) and FIO_2_ on V̇O_2_ (*F*_1.861,16.74_ = 11.76, *p* = 0.0008) and an interaction effect of work rate and FIO_2_ on V̇O_2_ (*F*_4.492,40.43_ = 21.01, *p* < 0.0001). V̇O_2_ was lower in hypoxia compared to normoxia and hyperoxia at 100 W, but otherwise there were no differences between the FIO_2_ conditions at submaximal work rates. We observed a main effect of work rate (*F*_1.649,14.84_ = 398.8, *p* < 0.0001) and an interaction effect of work rate and FIO_2_ on Q̇ (*F*_4.094,36.85_ = 5.359, *p* = 0.002), but not a main effect of FIO_2_ on Q̇ (*F*_1.999,17.99_ = 2.431, *p* = 0.116). At submaximal work rates, Q̇ was higher in hypoxia relative to normoxia (170 W and 275 W) and hyperoxia (170, 205, and 275 W), and there were no differences in Q̇ between normoxia and hyperoxia. We observed main effects of work rate (*F*_2.965,26.68_ = 644.4, *p* < 0.0001) and FIO_2_ (*F*_1.696,15.26_ = 17.39, *p* = 0.0002) on HR, and an interaction effect of work rate and FIO_2_ on HR (*F*_4.417,39.75_ = 12.29, *p* < 0.0001). At submaximal work rates, HR was higher in hypoxia compared to hyperoxia and normoxia across all work rates, and HR was higher in hyperoxia compared to normoxia at 240 W. Maximal HR (HR_max_) was lower in hypoxia relative to both normoxia and hyperoxia and similar between normoxia and hyperoxia. We observed a main effect of work rate (*F*_3.456,31.10_ = 140.8, *p* < 0.0001), but not of FIO_2_ (*F*_1.892,17.03_ = 0.487, *p* = 0.613) or an interaction effect of work rate and FIO_2_ on SV (*F*_4.021,36.19_ = 0.75, *p* = 0.563). We observed main effects of work rate (*F*_3.723,33.50_ = 147.8, *p* < 0.0001) and FIO_2_ (*F*_1.999,17.99_ = 5.75, *p* = 0.012) on C(a‐⊽)O_2_ and an interaction effect of work rate and FIO_2_ on C(a‐⊽)O_2_ (*F*_4.482,40.34_ = 3.11, *p* = 0.021). At submaximal work rates, C(a‐⊽)O_2_ was lower in hypoxia compared to normoxia (100 135, 170, 240, and 275 W) and relative to hyperoxia (100, 170, 240, and 275 W), whereas there were no differences in C(a‐⊽)O_2_ between normoxia and hyperoxia. We observed main effects of work rate (*F*_3.644,32.80_ = 52.04, *p* < 0.0001) and FIO_2_ (*F*_1.096,9.866_ = 194, *p* < 0.0001) on SpO_2_ and an interaction effect of work rate and FIO_2_ on SpO_2_ (*F*_3.659,32.93_ = 42.80, *p* < 0.0001). SpO_2_ was lower in hypoxia relative to both normoxia and hyperoxia across all submaximal work rates, while there were no differences in SpO_2_ between normoxia and hyperoxia at submaximal work rates. We observed main effects of work rate (*F*_3.377,30.39_ = 40.17, *p* < 0.0001) and FIO_2_ (*F*_1.669,15.02_ = 81.18, *p* < 0.0001) on CaO_2_ and an interaction effect of work rate and FIO_2_ on CaO_2_ (*F*_3.575,32.17_ = 42.80, *p* < 0.0001). CaO_2_ was lower in hypoxia relative to both normoxia and hyperoxia across all work rates, while there were no differences in CaO_2_ between normoxia and hyperoxia at submaximal work rates. We observed main effects of work rate (*F*_3.000,27.00_ = 135.5, *p* < 0.0001) and FIO_2_ (*F*_1.977,17.79_ = 4.54, *p* = 0.026) on C⊽O_2_ and an interaction effect of work rate and FIO_2_ on C⊽O_2_ (*F*_4.402,39.62_ = 4.42, *p* = 0.004). At submaximal work rates, C⊽O_2_ was lower in hypoxia relative to normoxia (205, 240, and 275 W), and to hyperoxia (205, 240, and 275 W), and there were no differences in C⊽O_2_ between normoxia and hyperoxia. We observed main effects of work rate (*F*_1.932,17.39_ = 487.1, *p* < 0.0001) and FIO_2_ (*F*_1.857,16.71_ = 7.27, *p* = 0.006) on Q̇aO_2_ and an interaction effect of work rate and FIO_2_ on Q̇aO_2_ (*F*_5.067,45.61_ = 19.35, *p* < 0.0001). Q̇aO_2_ was lower in hypoxia compared to normoxia and hyperoxia at submaximal work rates (240 and 275 W), while there were no differences in Q̇aO_2_ between normoxia and hyperoxia at submaximal work rates. We observed a main effect of work rate (*F*_3.086,27.77_ = 122.4, *p* < 0.0001) and an interaction effect of work rate and FIO_2_ on O_2_ extraction fraction (F_4.219,37.97_ = 2.883, *p* = 0.033), but not a main effect of FIO_2_ on O_2_ extraction fraction (*F*_1.948,17.53_ = 0.550, *p* = 0.582). O_2_ extraction fraction was higher in hypoxia relative to normoxia and hyperoxia at 240 W. No differences in O_2_ extraction fraction were observed at maximal work rates or between normoxia and hyperoxia at any work rate.

### 3.2 Effects of acutely varied FIO_2_ on convective and diffusive components of O_2_ transport during maximal exercise

**FIGURE 2:**
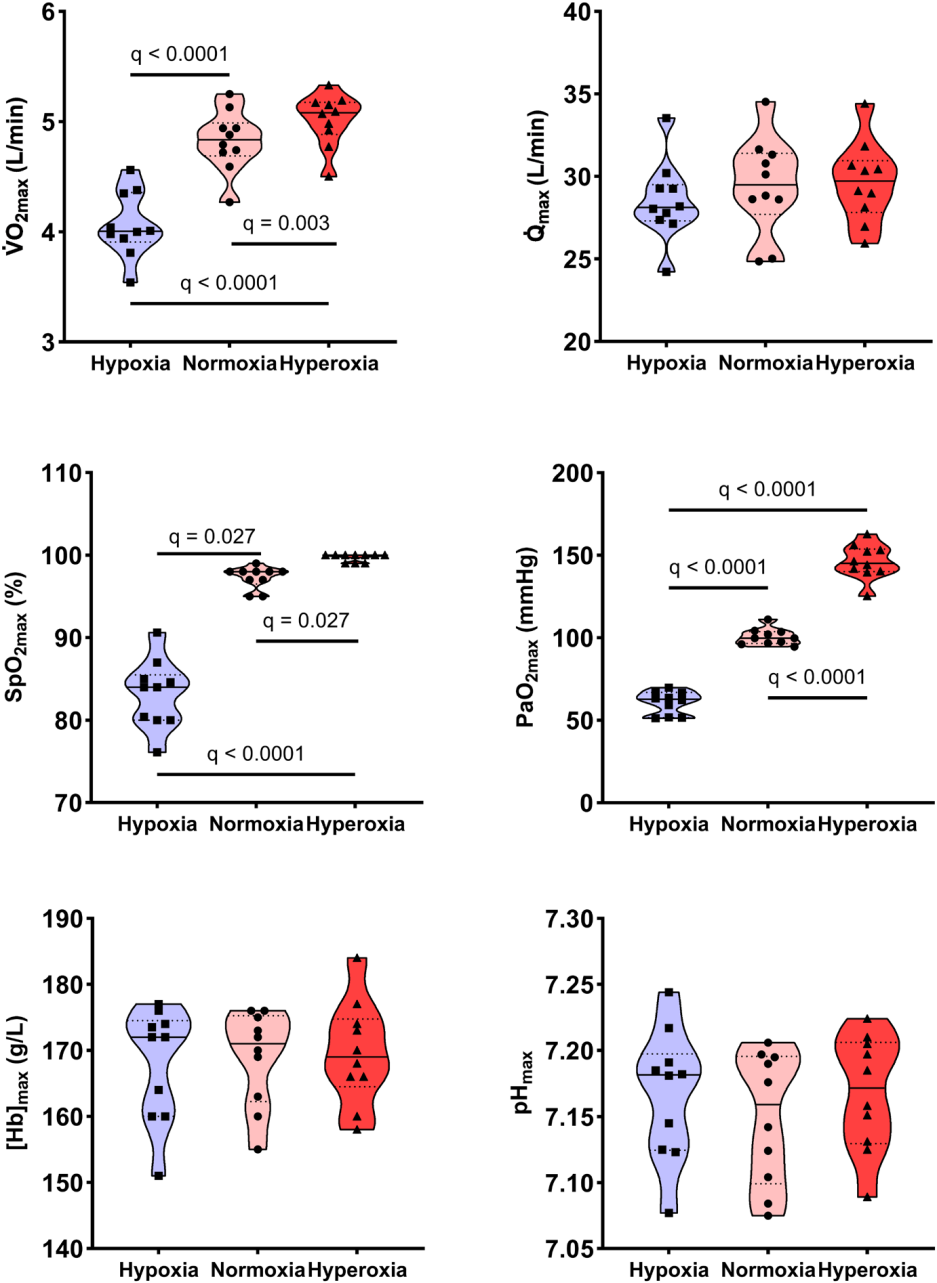
Maximal measured values of O_2_ transport. To illustrate both individual and group responses in the parameters used for calculating the convective and diffusive components of O_2_ transport, Figure 2 integrates nonparametric graphical representation with parametric statistical analysis. Full lines indicate the median, while dotted lines represent the 25% and 75% quartiles for each condition, *n* = 10. See Table 1 for abbreviations.

**FIGURE 3:**
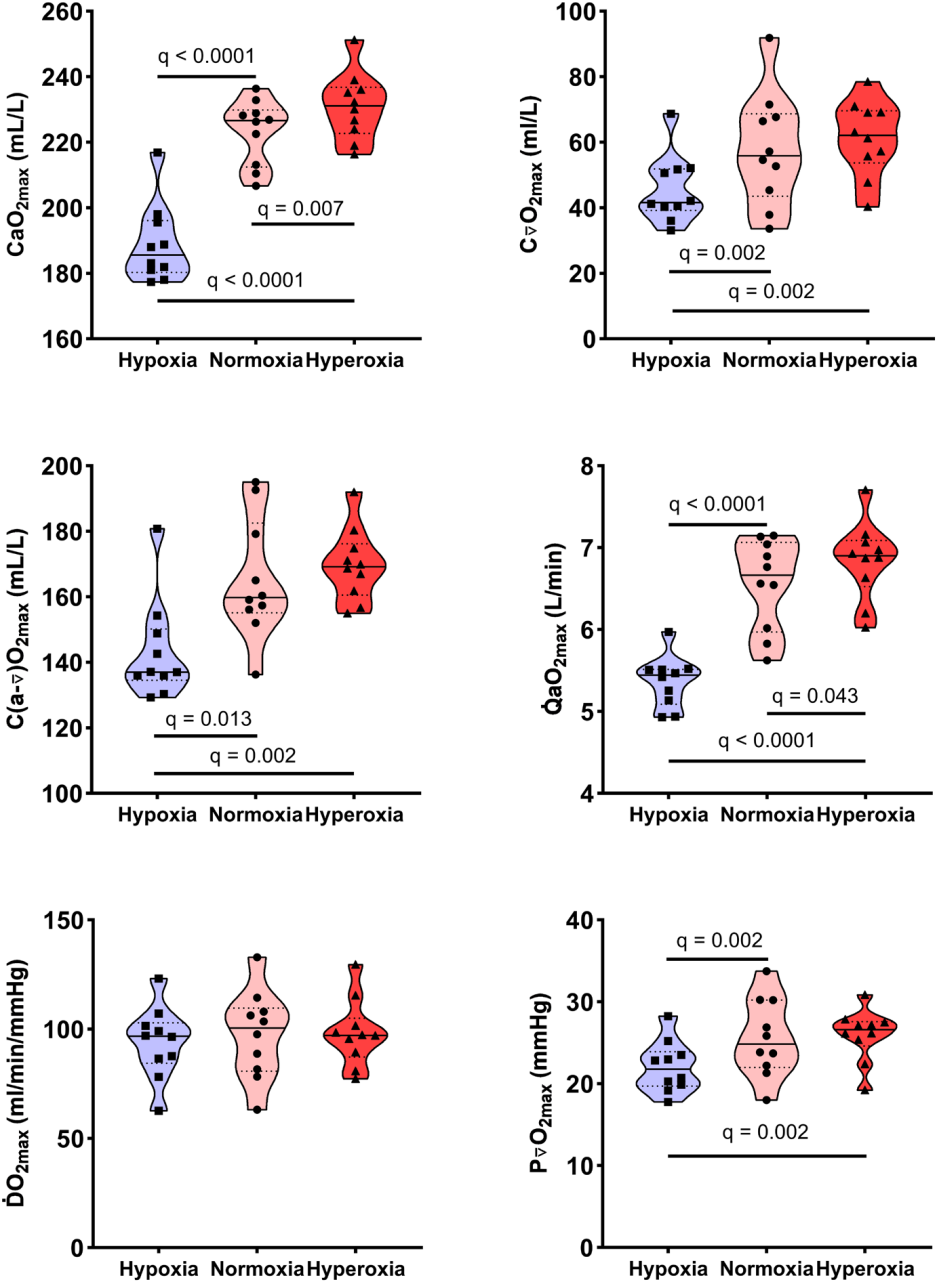
Maximal calculated values of convective and diffusive O_2_ transport. To illustrate both individual and group responses in the calculated parameters depicting the convective and diffusive components of O_2_ transport, Figure 3 integrates nonparametric graphical representation with parametric statistical analysis. Full lines indicate median while dotted lines represent the 25% and 75% quartiles for each group (*n* = 10). See Table 1 for abbreviations.

**FIGURE 4:**
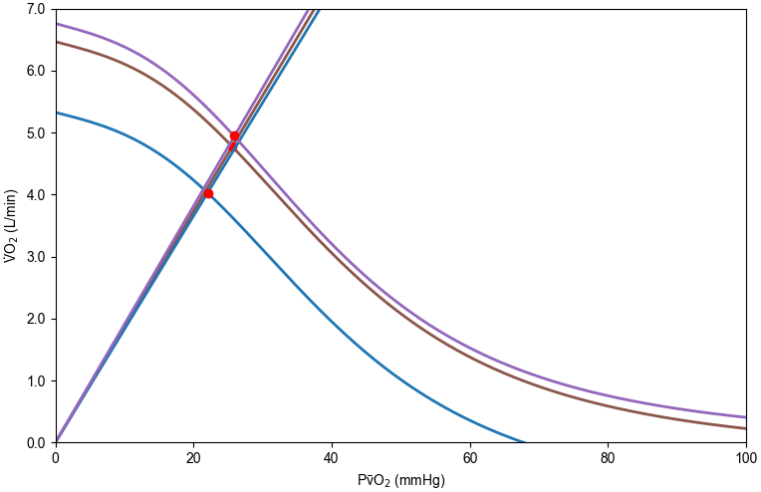
The Wagner diagram based on mean values of the convective and diffusive components of O_2_ transport determined in normoxia (brown), hypoxia (blue) and hyperoxia (purple) *n* = 10. The Fick principle [V̇O_2_ = *Q̇* × (CaO_2_ − C⊽O_2_)] is depicted by the curved line and Fick's law of diffusion (V̇O_2_ = ḊO_2_ × k × P⊽O_2_) by the straight line. The point, at which the lines intersect, represents V̇O_2max_. See Table 1 for abbreviations. V̇O_2_ = O_2_ uptake, k = diffusion constant. The figure was produced using the Helsinki O_2_ Pathway Tool (https://hula.fi/EP2/HO2PT) (Rissanen et al., 2024).

Table 1 as well as Figures 2 and 3 present key performance and mean maximal values of convective and diffusive components of O_2_ transport. Figure 4 illustrates the interplay of the convective and diffusive components of O_2_ transport in determining V̇O_2max_. *W*_max_ in hypoxia was lower compared to normoxia and hyperoxia, and *W*_max_ in hyperoxia was higher compared to normoxia. PaO_2max_ in hypoxia was lower compared to normoxia and hyperoxia, and PaO_2max_ in hyperoxia was higher compared to normoxia. V̇O_2max_ in hypoxia was lower compared to normoxia and hyperoxia, and V̇O_2max_ in hyperoxia was higher compared to normoxia. *Q̇*_max_ was similar across all three conditions. SpO_2max_ was lower in hypoxia relative to both normoxia and hyperoxia, and SpO_2max_ in hyperoxia was higher compared to normoxia. [Hb]_max_ and pH_max_ were similar across all three FIO_2_ conditions. *W*_VT1_ was different between the FIO_2_ conditions (*F*_1.908,17.71_ = 51.30, *p* < 0.0001). W_VT1_ in hypoxia (204 [190–218] *W*) was lower compared to normoxia (260 [241–279] *W*, *q* < 0.0001) and hyperoxia (273 [250–295] *W*, *q* < 0.0001), and W_VT1_ in hyperoxia was higher compared to normoxia (*q* = 0.029).

**TABLE 1:** Maximal values of convective and diffusive components of O_2_ transport.

	Hypoxia	Normoxia	Hyperoxia	*F* value	Main effect of FIO_2_ (*p* value)	*q* value (Normoxia vs. hypoxia)	*q* value (Normoxia vs. hyperoxia)	*q* value (hypoxia vs. hyperoxia)
Mean or median maximal values used to calculate the convective and diffusive components of O_2_ transport								
*W*_max_ (W), mean (95% CI)	338 (319–357)	399 (379–420)	413 (388–438)	(1.13, 10.15) = 93.8	<0.0001	<0.0001	0.004	<0.0001
V̇O_2max_ (L/min), mean (95% CI)	4.06 (3.85–4.27)	4.83 (4.63–5.02)	5.02 (4.85–5.19)	(1.59, 14.34) = 134.9	<0.0001	<0.0001	0.003	<0.0001
*Q̇*_max_ (L/min), mean (95% CI)	28.5 (26.8–30.2)	29.4 (27.3–31.6)	29.7 (27.9–31.4)	(1.88,16.96) = 1.279	0.302	‐	‐	‐
SpO_2max_ (%), median (IQR)	84 (80–86)	98 (97–98)	100 (99–100)	‐	< 0.0001	0.027	0.027	<0.0001
[Hb]_max_ (g/L), mean (95% CI)	168 (162–174)	169 (164–174)	170 (164–175)	(1.82,16.41) = 0.455	0.625	‐	‐	‐
PaO_2max_ (mmHg), mean (95% CI)	61 (56–66)	101 (97–104)	146 (139–154)	(1.62,14.58) = 381.4	<0.0001	<0.0001	<0.0001	<0.0001
pH_max_, mean (95% CI)	7.17 (7.13–7.20)	7.15 (7.11–7.19)	7.17 (7.14–7.20)	(1.52,13.68) = 0.588	0.524	‐	‐	‐
Mean or median maximal calculated values of the convective and diffusive components of O_2_ transport								
CaO_2max_ (mL/L), mean (95% CI)	189 (180–198)	223 (216–230)	231 (224–238)	(1.63,14.63) = 133.9	<0.0001	<0.0001	0.007	<0.0001
Q̇aO_2max_ (L/min), mean (95% CI)	5.37 (5.14–5.59)	6.56 (6.16–6.95)	6.84 (6.50–7.18)	(1.94,17.45) = 50.95	<0.0001	<0.0001	0.043	<0.0001
C⊽O_2max_ (mL/L), mean (95% CI)	46 (38–53)	58 (46–70)	61 (53–69)	(1.82,16.36) = 9.362	0.002	0.002	0.155	0.002
C(a‐⊽)O_2max_ (mL/L), median (IQR)	137 (135–150)	160 (155–183)	169 (161–176)	‐	0.003	0.013	>0.130	0.002
ḊO_2max_ (mL/min/mmHg), mean (95% CI)	94 (82–106)	98 (83–112)	98 (87–109)	(1.84,16.56) = 0.7201	0.490	‐	‐	‐
P⊽O_2max_ (mmHg), mean (95% CI)	22 (20–24)	26 (22–29)	26 (24–28)	(1.89,17.01) = 10.19	0.001	0.002	0.259	0.002

*Note*: Values are reported as mean (95% CI) for normally distributed variables and as median (IQR) for nonnormally distributed variables; *n* = 10.

Abbreviations: [Hb]_max_, hemoglobin concentration at maximal exercise; C(a‐⊽)O_2max_, arterial–venous O_2_ content difference at maximal exercise; C⊽O_2max_, mixed venous O_2_ content at maximal exercise; CaO_2max_, arterial O_2_ content at maximal exercise; ḊO_2max_, diffusive O_2_ conductance at maximal exercise; P⊽O_2max_, partial mixed venous O_2_ pressure at maximal exercise; PaO_2max_, partial arterial O_2_ pressure at maximal exercise; pH_max_, pH at maximal exercise; Q̇aO_2max_, convective O_2_ delivery at maximal exercise; Q̇_max_, maximal cardiac output; SpO_2max_, arterial O_2_ saturation estimated with pulse oximetry at maximal exercise; V̇O_2max_, maximal O_2_ uptake; *W*_max_, Maximal work rate.

CaO_2max_ in hypoxia was lower compared to normoxia and hyperoxia, and CaO_2max_ in hyperoxia was higher compared to normoxia. C⊽O_2max_ was lower in hypoxia compared to normoxia and hyperoxia, and similar between normoxia and hyperoxia. C(a‐⊽)O_2max_ was lower in hypoxia compared to normoxia and hyperoxia, but did not differ between normoxia and hyperoxia. Q̇aO_2max_ was lower in hypoxia compared to both normoxia and hyperoxia, and Q̇aO_2max_ was higher in hyperoxia relative to normoxia. ḊO_2max_ was similar across all FIO_2_ conditions. P⊽O_2max_ was lower in hypoxia compared to both normoxia and hyperoxia, and similar between normoxia and hyperoxia.

### 3.3 Effects of acutely varied FIO_2_ on muscle (de)oxygenation responses

**FIGURE 5:**
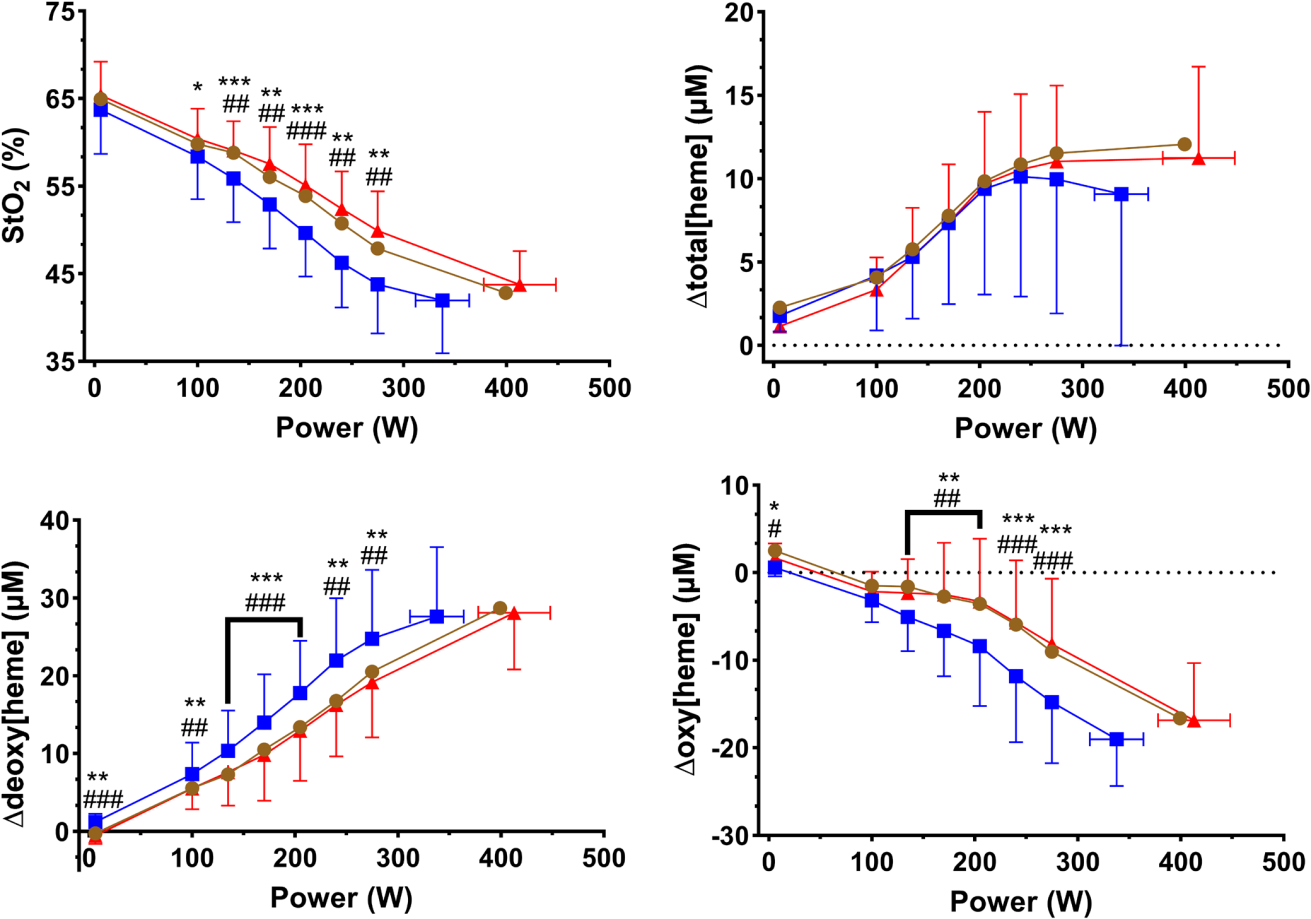
Work rate‐wise muscle (*vastus lateralis*) oxygenation responses to exercise in normoxia (brown circles), hypoxia (blue squares), and hyperoxia (red triangles). Data are presented as mean ± SD, *n* = 10. StO_2_ = tissue saturation index, ∆total[heme] = change in total hemo‐ and myoglobin from unloaded cycling, ∆deoxy[heme] = change in deoxygenated hemo‐ and myoglobin from unloaded cycling, ∆oxy[heme] = change in oxygenated hemo‐ and myoglobin from unloaded cycling. * Significant difference between hypoxia and normoxia, *q* < 0.05, **q < 0.01, ***q < 0.001. ^#^Significant difference between hypoxia and hyperoxia, *q* < 0.05, ^##^ *q* < 0.01, ^###^ *q* < 0.001.

No differences were observed in any of the NIRS metrics at rest before switching to the experimental FIO_2_ condition (*F*_1.345,12.10_ = 0.611, *p* = 0.496 for StO_2_, *F*_1.919,17.27_ = 0.172, *p* = 0.835 for ∆oxy[heme], *F*_1.759,15.83_ = 1.012, *p* = 0.376 for ∆deoxy[heme], *F*_1.931,17.38_ = 0.553, *p* = 0.579 for ∆total[heme]). We observed main effects of work rate (*F*_1.899,17.09_ = 68.46, *p* < 0.0001) and FIO_2_ (*F*_1.765,15.88_ = 6.68, *p* = 0.095) on StO_2_, as well as an interaction effect of work rate and FIO_2_ on StO_2_ (*F*_3.648,32.83_ = 6.52, *p* = 0.001). StO_2_ was similar during unloaded cycling before switching to the experimental FIO_2_ condition (*F*_1.834,16.50_ = 0.257, *p* = 0.758). Figure 5 displays work rate‐wise responses in muscle oxygenation. At submaximal work rates, StO_2_ was 1–4%‐units lower in hypoxia relative to normoxia from 100 to 275 W, and 3%–6%‐units lower relative to hyperoxia from 135 W to 275 W. We observed main effects of work rate (*F*_1.549,13.94_ = 43.57, *p* < 0.0001) and FIO_2_ (*F*_1.990,17.91_ = 11.02, *p* = 0.001) on ∆oxy[heme], but not an interaction effect of work rate and FIO_2_ on StO_2_ (*F*_3.229,29.06_ = 2.46, *p* = 0.078). ∆Oxy[heme] was lower in hypoxia compared to normoxia and hyperoxia during unloaded cycling. From 135 to 275 W, ∆oxy[heme] was 3–6 μM lower in hypoxia compared to normoxia and 3–7 μM lower in hypoxia compared to hyperoxia, while there were no differences between normoxia and hyperoxia in ∆oxy[heme]. We observed main effects of work rate (*F*_2.149,19.34_ = 59.90, *p* < 0.0001) and FIO_2_ (*F*_1.448,13.03_ = 10.11, *p* = 0.004) on ∆deoxy[heme], as well as an interaction effect of work rate and FIO_2_ on ∆deoxy[heme] (*F*_2.321,20.89_ = 3.82, *p* = 0.033). ∆Deoxy[heme] was higher in hypoxia compared to normoxia and hyperoxia during unloaded cycling. From 100 to 275 W, ∆deoxy[heme] was 2–5 μM higher in hypoxia compared to normoxia and 2–6 μM higher compared to hyperoxia, while there were no differences between normoxia and hyperoxia in ∆deoxy[heme]. We observed a main effect of work rate on ∆total[heme] (*F*_2.183,19.65_ = 11.77, *p* = 0.0003), but no main effect of FIO_2_ (*F*_1.867,16.81_ = 0.830, *p* = 0.446) or interaction effect of work rate and FIO_2_ (*F*_2.345,21.10_ = 0.521, *p* = 0.630) on ∆total[heme]. The NIRS metrics at maximal work rates were similar across the three FIO_2_ conditions.

**FIGURE 6:**
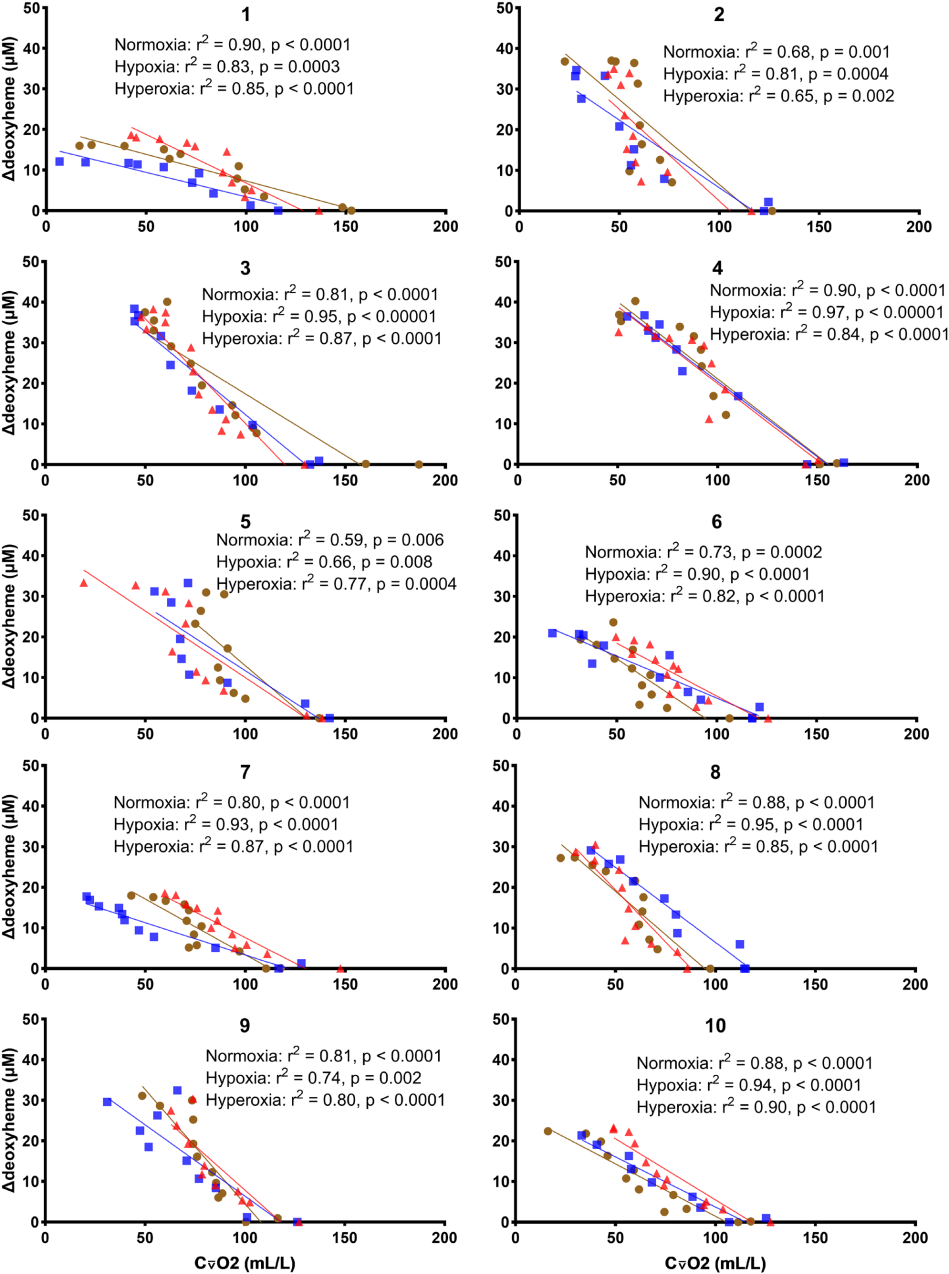
Individual relationships between whole‐body estimated mixed venous O_2_ content (C⊽O_2_) and the change in deoxygenated hemo‐ and myoglobin (∆deoxy[heme]) in 10 participants, during an incremental exercise test to exhaustion in normoxia (brown circles), hypoxia (blue squares), and hyperoxia (red triangles).

As depicted in Figure 6, for each individual in each FIO_2_ condition, a negative linear relationship between ∆deoxy[heme] and C⊽O_2_ was observed, but the slopes showed marked inter‐individual variation (*F*_9,101_ = 4.96, *p* < 0.0001; *F*_9,81_ = 6.55, *p* < 0.001; *F*_9,104_ = 3.52, *p* = 0.001 for the individual slopes in normoxia, hypoxia, and hyperoxia, respectively).

## 4 DISCUSSION

This study investigated central and peripheral responses of the O_2_ transport cascade non‐invasively with novel computational approaches at submaximal and maximal work rates in normoxia, acute moderate hypoxia and hyperoxia in Tier 3 (highly trained/national level) and Tier 4 (elite/international level) endurance athletes. Measured components of O_2_ transport are displayed in Figure 2 and calculated components in Figure 3. At submaximal work rates, both central and peripheral compensatory mechanisms maintained V̇O_2_ despite altered FIO_2_. At the central level, HR and Q̇ were higher in hypoxia at submaximal work rates and consequently Q̇aO_2_ was maintained at a similar level in hypoxia compared to normoxia up to 205 W, corresponding to 101% of W_VT1_ in hypoxia and 79% of W_VT1_ in normoxia, but at higher work rates Q̇aO_2_ was lower in hypoxia. Lower C⊽O_2_ and higher O_2_ extraction fraction together with higher ∆deoxy[heme] and lower StO_2_ at submaximal work rates in hypoxia suggest peripheral compensatory mechanisms reflecting altered matching of O_2_ transport to O_2_ utilization. At maximal exercise, these peripheral compensatory mechanisms reached their limits, as evidenced by similar *Q̇*_max_, ḊO_2max_ and muscle oxygenation at maximal work rate in all three FIO_2_ conditions. Consequently, V̇O_2max_ was lower in hypoxia and higher in hyperoxia compared to normoxia due to changes in Q̇aO_2max_ at the central level. Furthermore, lower Q̇aO_2max_ in acute moderate hypoxia resulted in reduced O_2_ diffusive driving pressure at the microvasculature at maximal exercise compared to normoxia and hyperoxia.

### 4.1 Effects of acutely varied FIO_2_ on O_2_ transport during submaximal exercise

We did not observe meaningful differences in V̇O_2_ at submaximal work rates between the FIO_2_ conditions. This aligns with previous findings of unaltered V̇O_2_ at submaximal work rates in moderate hypoxia (Wehrlin & Hallén, 2006), but contrasts with observations of increased V̇O_2_ in hyperoxia relative to hypoxia at submaximal work rates (Ulrich et al., 2017). However, the observations of increased V̇O_2_ in hyperoxia at submaximal work rates have attracted methodological criticism, especially in relation to the application of the Haldane‐ or Eschenbacher transformation in hyperoxic conditions (Eschenbacher, 2017). Particularly, the utilization of the Haldane transformation can yield incorrect V̇O_2_ values when high O_2_ concentrations above 0.40 FIO_2_ are utilized. In our study, 0.298 FIO_2_ was utilized, perhaps alleviating these discrepancies and therefore increasing the validity of the measured V̇O_2_ values.

At submaximal intensities, CaO_2_ was lower in hypoxia compared to normoxia and hyperoxia and despite lower C⊽O_2_, C(a‐⊽)O_2_ was also lower in hypoxia. Lower C(a‐⊽)O_2_ at submaximal work rates was observed along with higher Q̇, which was achieved by elevated HR at submaximal work rates, with no changes in SV. A similar compensatory mechanism to maintain Q̇aO_2_ in acute hypoxia has been previously observed (Calbet et al., 2003; Peltonen et al., 2001). However, while elevated Q̇ compensated initially for lower CaO_2_ fully, at higher submaximal intensities Q̇aO_2_ was lower in acute hypoxia compared to normoxia and hyperoxia. As V̇O_2_ was not yet compromised at these intensities, other compensatory mechanisms for V̇O_2_ must have been present. We observed increased peripheral O_2_ extraction in hypoxia relative to normoxia and hyperoxia concurrently with decreased Q̇aO_2_. However, whether this is due to increased ḊO_2_ or a steeper decline in PmitO_2_ cannot be assessed in the present setting. As we observed a similar trend in ∆total[heme], a potential proxy for ḊO_2_ (Barstow, 2019; Goulding et al., 2021), in hypoxia compared to normoxia and hyperoxia, a steeper decline in PmitO_2_ should be considered. Another potential contributing mechanism is increased muscle blood flow due to hypoxemia (Rowell et al., 1986), but this is countered by observations of similar proportions of leg blood flow and Q̇ in hypoxia and normoxia (Calbet et al., 2003; Hartley et al., 1973). In alignment with previous findings (Peltonen et al., 2001), we observed no alterations in Q̇ and HR at submaximal intensities between normoxic and hyperoxic conditions.

### 4.2 Effects of acutely varied FIO_2_ on convective and diffusive components of O_2_ transport during maximal exercise

Compared to normoxia, V̇O_2max_ was 16% lower in moderate acute hypoxia and 4% higher in acute moderate hyperoxia. The magnitude of V̇O_2max_ change from normoxia to hypoxia is similar to previous observations in endurance‐trained subjects at similar levels of FIO_2_ (Bourdillon et al., 2009; Ferretti et al., 1997; Wehrlin & Hallén, 2006). The changes in V̇O_2max_ were due to changes in both central and peripheral steps of O_2_ transport. Compared to normoxia, CaO_2max_ was 15% lower in hypoxia and 4% higher in hyperoxia due to alterations in both SpO_2max_ and PaO_2max_. Consequently, Q̇aO_2max_ was 18% lower in hypoxia and 4% higher in hyperoxia compared to normoxia. At the peripheral level, ḊO_2max_ was unchanged, but the rate of O_2_ transport was altered due to changes in O_2_ diffusive driving pressure. The changes in V̇O_2max_ were reflected in *W*_max_, which was 15% lower in hypoxia, and 4% higher in hyperoxia relative to normoxia. Previously, studies utilizing 0.15–0.16 FIO_2_ and 0.30–0.32 FIO_2_ have reported 13–16% lower *W*_max_ in hypoxia and 5%–6% higher *W*_max_ in hyperoxia compared to normoxia in endurance‐trained athletes (Ferretti et al., 1997; Peltonen et al., 2001).

*Q̇*_max_ was not affected by moderately altered FIO_2_. While altered *Q̇*_max_ in moderately altered FIO_2_ has been observed (Peltonen et al., 2001), this study supports the prevailing consensus that decreased *Q̇*_max_ is only observed at severe hypoxia (Calbet et al., 2003) or hyperoxia (Sperlich et al., 2016). Due to decreased CaO_2max_ and unchanged *Q̇*_max_, Q̇aO_2max_ was lower in hypoxia relative to both normoxia and hyperoxia. As previously observed (Roca et al., 1989), the peripheral diffusive component of O_2_ transport, ḊO_2max_, is not affected by moderately altered FIO_2_. However, P⊽O_2max_ was 14% and 15% lower in hypoxia compared to both normoxia and hyperoxia respectively. Assuming unaltered k in Equation 5, this reflects a decrease in the difference between PcapO_2_ and PmitO_2_, indicating reduced O_2_ diffusive driving pressure, which from the whole‐body perspective is a result of the decrease of Q̇aO_2max_ along with decreasing FIO_2_. Hence, the rate of peripheral O_2_ flow from capillaries to mitochondria was compromised in acute moderate hypoxia due to reduced Q̇aO_2max_ and subsequently reduced O_2_ diffusive driving pressure. In acute moderate hyperoxia, no significant difference was observed in either ḊO_2max_ or P⊽O_2max_ relative to normoxia. However, as PaO_2max_ was 45% higher in hyperoxia and P⊽O_2max_ was similar to normoxia, likely O_2_ diffusive driving pressure at the microvascular level was also higher in hyperoxia relative to normoxia.

Unchanged ḊO_2max_ between the FIO_2_ conditions indicates that the mitochondrial capacity to utilize O_2_ was not a limiting factor of V̇O_2max_ in our study population of Tier 3 and 4 endurance athletes unlike in sedentary individuals whose limitation shifts from mitochondrial oxidative capacity to O_2_ transport with endurance training (Broxterman et al., 2024). Furthermore, since ḊO_2max_ was unchanged, the underlying assumption of PmitO_2_ made in Equation 5 is justified. The interplay between the convective and diffusive components of O_2_ transport, combining the Fick principle (Equation 3) and Fick's law (Equation 5) into a single diagram, is illustrated in Figure 4.

Both P⊽O_2max_ and C⊽O_2max_ were lower in hypoxia relative to normoxia and hyperoxia. CaO_2max_ decreased to a greater extent compared to C⊽O_2max_ in hypoxia; consequently C(a‐⊽)O_2max_ was lower in hypoxia compared to normoxia and hyperoxia. Crucially, in this instance, C(a‐⊽)O_2max_ decreased due to changes in convective, often termed central, alterations in O_2_ transport. This must be stressed, as C(a‐⊽)O_2max_ is often used as a proxy for peripheral O_2_ transport (Gifford et al., 2024; Poole & Musch, 2008), but as demonstrated here, it can be affected by both central and peripheral components of O_2_ transport.

### 4.3 Effects of acutely varied FIO_2_ on muscle oxygenation responses to exercise

The findings of this study suggest that, in Tier 3 and 4 endurance athletes, acute moderate hypoxia alters muscle oxygenation at submaximal intensities, thus reflecting altered matching of O_2_ transport to utilization at the local level. This parallels the observations made at the whole‐body level in terms of decreased CaO_2_ and increased C⊽O_2_. At maximal intensity, muscle oxygenation was affected to a similar extent in altered conditions of FIO_2_. Crucially, in hypoxia, alterations to muscle oxygenation develop at a lower work rate, and thus, while muscle oxygenation is altered to a similar extent at exhaustion, this corresponds to a significantly lower work rate. At maximal intensity, the observed differences between the FIO_2_ conditions in whole‐body O_2_ transport were not reflected in local muscle oxygenation.

In parallel with no observed changes in ḊO_2max_, we observed no changes in ∆total[heme] between the FIO_2_ conditions. Changes in ∆total[heme] during exercise reflect changes in microvascular hematocrit volume (Davis & Barstow, 2013). During exercise, muscle ḊO_2_ is increased via recruitment of additional capillary surface, an increased proportion of capillaries contributing to blood‐myocyte flux, and elevations in capillary hematocrit (Poole et al., 2013). Hence, ∆total[heme] during exercise potentially reflects muscle ḊO_2_ (Barstow, 2019). Observations of higher ∆total[heme] amplitude in individuals with a higher V̇O_2max_ (Boone, Barstow, et al., 2016), coupled with the observed positive linear correlation between leg muscle ḊO_2max_ and V̇O_2max_ (Skattebo et al., 2020) support the idea that ∆total[heme] amplitude and ḊO_2max_ might be linked. However, to robustly establish the connection between ∆total[heme] and ḊO_2max_, studies directly manipulating ḊO_2max_, for example via manipulating additional capillary surface recruitment or capillary hematocrit volume of working muscles, are needed. We observed similar ∆total[heme] values at both submaximal and maximal intensities in all three FIO_2_ conditions, potentially indicating that *vastus lateralis* ḊO_2_ increased at a similar rate despite alterations in FIO_2_. We did not examine the kinetics of ∆total[heme], but previously, a plateau in ∆total[heme] has been suggested to indicate that ḊO_2_ at the measurement site has reached its ceiling (Goulding et al., 2021). However, as different working muscles can differ markedly in terms of ∆total[heme] kinetics, local ∆total[heme] responses might not be indicative of whole‐body ḊO_2_ responses (Goulding et al., 2021).

∆Deoxy[heme] has been suggested as a surrogate for C(a‐⊽)O_2_ at the muscle level (Barstow, 2019; Grassi & Quaresima, 2016; Murias et al., 2013). However, our results question this interpretation. In Figure 1, an increase in C(a‐⊽)O_2_ was observed as work rate increased, and in Figure 5, an increase in ∆deoxy[heme] was similarly observed as work rate increased. However, when FIO_2_ was decreased, C(a‐⊽)O_2_ decreased whereas ∆deoxy[heme] increased. Thus, changes in work rate and FIO_2_ elicited contradictory changes in C(a‐⊽)O_2_ and ∆deoxy[heme]. Contrastingly, C⊽O_2_ and ∆deoxy[heme] trends were better aligned with each other. A decrease in C⊽O_2_ was observed as work rate increased, and as FIO_2_ decreased. Simultaneously, ∆deoxy[heme] increased as work rate increased, and as FIO_2_ decreased. Previously, a negative linear correlation between ∆deoxy[heme] and C⊽O_2_ has been observed in animals (Sun et al., 2016). Similarly, we observed a negative linear correlation between C⊽O_2_ and ∆deoxy[heme] (Figure 6). In a similar vein, Martin‐Rincon et al. (2021) observed a strong negative linear correlation between invasively determined femoral O_2_ extraction and femoral venous O_2_ content when plotted against StO_2_. While at a group level, they did not observe differences between right and left leg or between sexes, their results also show marked interindividual variation in the O_2_ extraction versus StO_2_ relationship.

Consequently, both ∆deoxy[heme] and C⊽O_2_ reflect (im)balance between O_2_ transport and utilization. However, caution should be applied in using ∆deoxy[heme] as a surrogate for C⊽O_2_. While we observed strong correlations between the individual C⊽O_2_ and ∆deoxy[heme] values, and the correlations persisted at the group level, the individual slopes between C⊽O_2_ and ∆deoxy[heme] showed marked differences. This further highlights that ∆deoxy[heme] and C⊽O_2_ should not be used interchangeably. Concern has also been raised about the effects of ∆total[heme], skin and adipose tissue thickness on the relationship between ∆deoxy[heme] and C⊽O_2_ (Porcelli et al., 2023). However, in the present study, ∆total[heme] was similar in the different FIO_2_ conditions at both submaximal and maximal intensities. Further caution is warranted, as despite altered C⊽O_2max_ in hypoxia and hyperoxia, ∆deoxy[heme] was similar at maximal intensities across the FIO_2_ conditions.

In summary, the current study offers some key insights into the relationship between whole‐body and locally determined variables of O_2_ transport and utilization in endurance athletes. First, while at a whole‐body level P⊽O_2max_ was lower in hypoxia, StO_2_ was similar across all three FIO_2_ conditions at maximal exercise. This suggests that it cannot be used as a proxy for P⊽O_2max_ during whole‐body exercise (Manferdelli et al., 2023; Porcelli et al., 2023). Second, as ∆total[heme] at maximal exercise and ḊO_2max_ were similar across FIO_2_ conditions, and as there is a physiological rationale linking the two together, ∆total[heme] might be a suitable proxy for ḊO_2max_. To establish this link, experiments manipulating ḊO_2max_ are needed. Third, during submaximal exercise in hypoxia relative to normoxia and hyperoxia, concurrent observations of decreased whole‐body Q̇aO_2_ relative to V̇O_2_ and increased ∆deoxy[heme] suggest together a greater reliance on local O_2_ extraction. This opens avenues for further studies to examine whether NIRS kinetics during exercise can be used to discern the intensity at which PmitO_2_ reaches minimum values as well as how ḊO_2_ behaves in relation to exercise intensity.

### 4.4 Methodological considerations

The present study relied on non‐invasive determination of SV and thereby Q̇ with the PhysioFlow ICG device. The device has been validated against the direct Fick method from rest to maximal exercise (Charloux et al., 2000; Richard et al., 2001) and has also been shown to provide reliable group‐level data on SV from VT1 to maximal exercise in well‐trained athletes. In addition, compared to other non‐invasive methods (inert gas rebreathing and pulse contour analysis) determining SV and Q̇ during exercise, the PhysioFlow ICG device produced less physiologically implausible values (Siebenmann et al., 2015).

Mollard et al. (2010) compared SpO_2_ determined from earlobe against arterial O_2_ saturation during incremental exercise in normoxia and hypoxia (FIO_2_ 0.127) and found good agreement between the two methods (mean earlobe error of −0.69 and 95% CI of ±2.09%). However, earlobe SpO_2_ precision worsened when arterial O_2_ saturation fell below 75%. In the present study, a higher FIO_2_ was used during hypoxia, alleviating the potential problems caused by low arterial O_2_ saturation. In their meta‐analysis, Zavorsky et al. (2007) concluded that PaO_2_ can be determined from earlobe capillary samples at an acceptable accuracy (95% CI 1.9–2.8 mmHg) and that the accuracy is better at low PaO_2_ values and during exercise. Similarly, Mollard et al. (2010) concluded that the accuracy of earlobe determined PaO_2_ is better at low PaO_2_ values. Therefore, in the present study, the hyperoxic condition was the most likely to yield errors. Based on the study of Mollard et al. (2010) earlobe samples are more likely to under‐ than overestimate PaO_2_ values at high values. This would have led to the underestimation of Q̇aO_2max_ and consequently the overestimation of ḊO_2max_ in the hyperoxic condition. Our results indicate that this did not happen as ḊO_2max_ did not differ between the FIO_2_ conditions. Both Zavorsky et al. (2007) and Mollard et al. (Mollard et al., 2010) concluded that earlobe is an acceptable substitute for arterial pH.

The present study further relied on the validated non‐invasive methods to estimate the components of O_2_ transport. The convective and diffusive components of V̇O_2max_, as presented in the diagram Wagner (2011), were calculated with the Helsinki O_2_ Pathway Tool (Rissanen et al., 2024) based on non‐invasive whole‐body measurements. These calculations rest on several assumptions which are more extensively discussed elsewhere (Rissanen et al., 2024) and of course fully apply also to this study. First, PmitO_2_ was assumed to be close to zero (Richardson et al., 1995; Roca et al., 1989). Calculated ḊO_2max_ did not differ between the FIO_2_ conditions, supporting this assumption. Second, ḊO_2max_ was calculated based on whole‐body V̇O_2max_ and *Q̇*_max_, and these calculations are subject to error due to blood flow to non‐exercising tissues as well as non‐uniform inter‐ or intramuscular blood flow (Heinonen et al., 2015; Rissanen et al., 2024; Roca et al., 1989). Third, ḊO_2max_ was calculated by assuming that the constant k in Equation 5 equals 2, while both intra‐ and interindividual variation in k has been reported (Roca et al., 1989). Based on a group‐level sensitivity analysis presented by Rissanen et al. (Rissanen et al., 2024), random variation of constant k between 1.8 and 2.2 does not lead to a difference in ḊO_2max_ compared to a fixed value of 2.

Skattebo et al. (2020) combined data from studies utilizing gold standard invasive methods. The values we obtained in normoxia bear close resemblance to those obtained with invasive methods for subjects of a similar fitness status. Furthermore, the changes in the central and peripheral steps of O_2_ transport in response to hypoxia are similar to those made by Roca et al. (1989) in similar normoxic and hypoxic conditions. This supports the applicability of non‐invasive methods in estimating the individual components of O_2_ transport, and thus encourages the application of the Wagner diagram also beyond studies relying on invasive methods.

In the current study local NIRS measurements were compared to systemic determinants of O_2_ transport and utilization. However, two issues need to be considered in this regard: First, as considerable heterogeneity has been observed between NIRS responses of different muscle groups (Barstow, 2019; Goulding et al., 2021), the current findings only apply to the active vastus lateralis muscle. Second, although the NIRS signal is measured locally, all systemic adjustments of the O_2_ pathway occurring before the active muscle level also affect the NIRS responses. Barstow (2019) provides a more detailed description of methodological considerations relating to NIRS in the context of skeletal muscle research.

The present study was conducted with male endurance athletes. Only males were included in the study as we aimed to minimize the impact of adipose tissue thickness on the NIRS signal (Barstow, 2019). This is why the present results cannot be generalized to women. In this regard, we see an important knowledge gap needing filling by future studies, as at least we are not aware of studies comparing differences in exercise‐induced responses of convective and diffusive O_2_ transport between sexes in athletic individuals. Training status in particular influences the determinants of V̇O_2max_ and consequently responses to altered FIO_2_ (Bourdillon et al., 2009; Broxterman et al., 2024). The observations of this study should not be generalized to other populations. In addition, responses to hypoxia in terms of O_2_ transport and V̇O_2_ differ between acute and chronic exposure and between moderate and severe hypoxia (Calbet et al., 2003; Calbet et al., 2009). Furthermore, normobaric and hypobaric hypoxia can elicit different responses on determinants of O_2_ transport (Rosales et al., 2022).

## 5 CONCLUSIONS

Our study demonstrates how the different steps of O_2_ transport are affected by acute moderate hypoxia and hyperoxia in Tier 3 and 4 endurance athletes during submaximal and maximal whole‐body exercise. At submaximal work rates, V̇O_2_ was maintained despite altered FIO_2_ via both central and peripheral compensatory mechanisms. In acute moderate hypoxia central compensatory mechanisms partially preserved Q̇aO_2_, and peripheral compensatory mechanisms altered the matching of O_2_ transport and utilization. At maximal exercise, these compensatory mechanisms reached their limits. Consequently, V̇O_2max_ was lower in hypoxia and higher in hyperoxia due to changes in Q̇aO_2max_ and peripheral O_2_ diffusive driving pressure, whereas *Q̇*_max_, ḊO_2max_ and muscle oxygenation at maximal work rate were similar across the three FIO_2_ conditions. Finally, while at submaximal intensities muscle oxygenation reflected altered matching of Q̇aO_2_ to V̇O_2_, at maximal exercise altered whole‐body P⊽O_2max_ was not reflected by NIRS.

## 6 PERSPECTIVES AND SIGNIFICANCE

The present study depicts the interplay of the convective and diffusive components of O_2_ transport in endurance athletes in varied conditions of FIO_2_. In particular, we demonstrated how various non‐invasive methods can be combined with novel computational approaches for improved understanding of how all steps of O_2_ transport together determine V̇O_2max_. V̇O_2max_ is one of the most studied metrics in the field of physiology, and the proper understanding of its determinants is of significance in the field of health and disease, sports performance and environmental physiology. Therefore, we encourage the application of the approaches presented in this article in varied contexts. However, as this study was based solely on non‐invasive methodologies, future studies should also examine how the whole‐body and peripheral muscle oxygenation responses presented here relate to invasively determined components of O_2_ transport.

## AUTHOR CONTRIBUTIONS

Experimental measurements were performed at the Helsinki Sports and Exercise Medicine Clinic (HULA) laboratory in Helsinki, Finland. Elias Lehtonen, Dominique D. Gagnon, and Juha E. Peltonen contributed to the conception and design of the work. Elias Lehtonen, Antti‐Pekka E. Rissanen and Tom Mikkola contributed to data analysis. Elias Lehtonen performed the experiments and prepared figures for the manuscript. Elias Lehtonen, Dominique D. Gagnon, Antti‐Pekka E. Rissanen, and Juha E. Peltonen contributed to interpreting the results of experiments. Elias Lehtonen drafted the manuscript. Dominique D. Gagnon, Antti‐Pekka E. Rissanen, and Juha E. Peltonen edited and revised the manuscript. All authors approved the final version of the manuscript and agree to be accountable for all aspects of the work. All persons designated as authors qualify for authorship, and everyone qualified for authorship has been included as authors.

## FUNDING INFORMATION

This study was financially supported by the Ministry of Education and Culture, Finland (OKM/128/626/2021; OKM/39/626/2022; OKM/67/626/2023) and Urheiluopistosäätiö (12/10/2021).

## CONFLICT OF INTEREST STATEMENT

No conflict of interest, financial, or otherwise, are declared by the authors.

## ETHICS STATEMENT

None.

## Data Availability

Data will be made available upon reasonable request.

## References

[phy270639-bib-0001] Barstow, T. J. (2019). Understanding near infrared spectroscopy and its application to skeletal muscle research. Journal of Applied Physiology (1985), 126, 1360–1376. 10.1152/japplphysiol.00166.2018 30844336

[phy270639-bib-0002] Beaver, W. L. , Wasserman, K. , & Whipp, B. J. (1986). A new method for detecting anaerobic threshold by gas exchange. Journal of Applied Physiology, 60, 2020–2027. 3087938 10.1152/jappl.1986.60.6.2020

[phy270639-bib-0003] Benjamini, Y. , Krieger, A. M. , & Yekutieli, D. (2006). Adaptive linear step‐up procedures that control the false discovery rate. Biometrika, 93, 491–507.

[phy270639-bib-0004] Boone, J. , Barstow, T. J. , Celie, B. , Prieur, F. , & Bourgois, J. (2016). The interrelationship between muscle oxygenation, muscle activation, and pulmonary oxygen uptake to incremental ramp exercise: influence of aerobic fitness. Applied Physiology, Nutrition, and Metabolism, 41, 55–62. 10.1139/apnm-2015-0261 26701120

[phy270639-bib-0005] Boone, J. , Vandekerckhove, K. , Coomans, I. , Prieur, F. , & Bourgois, J. G. (2016). An integrated view on the oxygenation responses to incremental exercise at the brain, the locomotor and respiratory muscles. European Journal of Applied Physiology, 116, 2085–2102. 27613650 10.1007/s00421-016-3468-x

[phy270639-bib-0006] Bourdillon, N. , Mollard, P. , Letournel, M. , Beaudry, M. , & Richalet, J. P. (2009). Interaction between hypoxia and training on NIRS signal during exercise: contribution of a mathematical model. Respiratory Physiology & Neurobiology, 169, 50–61. 19712759 10.1016/j.resp.2009.08.011

[phy270639-bib-0007] Broxterman, R. M. , Wagner, P. D. , & Richardson, R. S. (2024). Endurance exercise training changes the limitation on muscle V̇o_2_max in normoxia from the capacity to utilize O_2_ to the capacity to transport O_2_ . The Journal of Physiology, 602, 445–459. 38048175 10.1113/JP285650 PMC10841684

[phy270639-bib-0008] Calbet, J. A. , Boushel, R. , Rådegran, G. , Søndergaard, H. , Wagner, P. D. , & Saltin, B. (2003). Determinants of maximal oxygen uptake in severe acute hypoxia. American Journal of Physiology. Regulatory, Integrative and Comparative Physiology, 284, R291–R303. 12388461 10.1152/ajpregu.00155.2002

[phy270639-bib-0009] Calbet, J. A. , Holmberg, H. C. , Rosdahl, H. , van Hall, G. , Jensen‐Urstad, M. , & Saltin, B. (2005). Why do arms extract less oxygen than legs during exercise? American Journal of Physiology. Regulatory, Integrative and Comparative Physiology, 289, R1448–R1458. 15919729 10.1152/ajpregu.00824.2004

[phy270639-bib-0010] Calbet, J. A. , Rådegran, G. , Boushel, R. , & Saltin, B. (2009). On the mechanisms that limit oxygen uptake during exercise in acute and chronic hypoxia: role of muscle mass. The Journal of Physiology, 587, 477–490. 19047206 10.1113/jphysiol.2008.162271 PMC2670057

[phy270639-bib-0011] Cardinale, D. A. , Larsen, F. J. , Jensen‐Urstad, M. , Rullman, E. , Søndergaard, H. , Morales‐Alamo, D. , Ekblom, B. , Calbet, J. A. L. , & Boushel, R. (2019). Muscle mass and inspired oxygen influence oxygen extraction at maximal exercise: Role of mitochondrial oxygen affinity. Acta Physiologica (Oxford, England), 225, e13110. 29863764 10.1111/apha.13110

[phy270639-bib-0012] Cardús, J. , Marrades, R. M. , Roca, J. , Barberà, J. A. , Diaz, O. , Masclans, J. R. , Rodriguez‐Roisin, R. , & Wagner, P. D. (1998). Effects of F(I)O_2_ on leg VO_2_ during cycle ergometry in sedentary subjects. Medicine and Science in Sports and Exercise, 30, 697–703. 9588611 10.1097/00005768-199805000-00009

[phy270639-bib-0013] Charloux, A. , Lonsdorfer‐Wolf, E. , Richard, R. , Lampert, E. , Oswald‐Mammosser, M. , Mettauer, B. , Geny, B. , & Lonsdorfer, J. (2000). A new impedance cardiograph device for the non‐invasive evaluation of cardiac output at rest and during exercise: comparison with the “direct” Fick method. European Journal of Applied Physiology, 82, 313–320. 10958374 10.1007/s004210000226

[phy270639-bib-0014] Davis, M. L. , & Barstow, T. J. (2013). Estimated contribution of hemoglobin and myoglobin to near infrared spectroscopy. Respiratory Physiology & Neurobiology, 186, 180–187. 23357615 10.1016/j.resp.2013.01.012

[phy270639-bib-0015] Eschenbacher, H. (2017). Cardiopulmonary exercise testing under hyperoxia: Is the Haldane‐transformation the correct approach? Respiration, 94, 71. 28478455 10.1159/000475505

[phy270639-bib-0016] Ferretti, G. , Moia, C. , Thomet, J. M. , & Kayser, B. (1997). The decrease of maximal oxygen consumption during hypoxia in man: a mirror image of the oxygen equilibrium curve. The Journal of Physiology, 498(Pt 1), 231–237. 9023781 10.1113/jphysiol.1997.sp021854 PMC1159247

[phy270639-bib-0017] Gifford, J. R. , Blackmon, C. , Hales, K. , Hinkle, L. J. , & Richards, S. (2024). Overdot and overline annotation must be understood to accurately interpret V.O2MAX physiology with the Fick formula. Frontiers in Physiology, 15, 1359119. 38444762 10.3389/fphys.2024.1359119 PMC10912163

[phy270639-bib-0018] Gifford, J. R. , Garten, R. S. , Nelson, A. D. , Trinity, J. D. , Layec, G. , Witman, M. A. , Weavil, J. C. , Mangum, T. , Hart, C. , Etheredge, C. , Jessop, J. , Bledsoe, A. , Morgan, D. E. , Wray, D. W. , Rossman, M. J. , & Richardson, R. S. (2016). Symmorphosis and skeletal muscle V̇o_2_ max: in vivo and in vitro measures reveal differing constraints in the exercise‐trained and untrained human. The Journal of Physiology, 594, 1741–1751. 26614395 10.1113/JP271229 PMC4799962

[phy270639-bib-0019] González‐Alonso, J. , Calbet, J. A. , Boushel, R. , Helge, J. W. , Søndergaard, H. , Munch‐Andersen, T. , van Hall, G. , Mortensen, S. P. , & Secher, N. H. (2015). Blood temperature and perfusion to exercising and non‐exercising human limbs. Experimental Physiology, 100, 1118–1131. 26268717 10.1113/EP085383 PMC5049637

[phy270639-bib-0020] Goulding, R. P. (2024). Re‐evaluating central versus peripheral contributions to maximal oxygen uptake: the role of muscle diffusive capacity. The Journal of Physiology, 602, 5391–5393. 39216088 10.1113/JP287378

[phy270639-bib-0021] Goulding, R. P. , Marwood, S. , Lei, T. H. , Okushima, D. , Poole, D. C. , Barstow, T. J. , Kondo, N. , & Koga, S. (2021). Dissociation between exercise intensity thresholds: mechanistic insights from supine exercise. American Journal of Physiology. Regulatory, Integrative and Comparative Physiology, 321, R712–R722. 34431402 10.1152/ajpregu.00096.2021

[phy270639-bib-0022] Grassi, B. , & Quaresima, V. (2016). Near‐infrared spectroscopy and skeletal muscle oxidative function in vivo in health and disease: a review from an exercise physiology perspective. Journal of Biomedical Optics, 21, 091313. 27443955 10.1117/1.JBO.21.9.091313

[phy270639-bib-0023] Hartley, L. H. , Vogel, J. A. , & Landowne, M. (1973). Central, femoral, and brachial circulation during exercise in hypoxia. Journal of Applied Physiology, 34, 87–90. 4572510 10.1152/jappl.1973.34.1.87

[phy270639-bib-0024] Heinonen, I. , Koga, S. , Kalliokoski, K. K. , Musch, T. I. , & Poole, D. C. (2015). Heterogeneity of muscle blood flow and metabolism: Influence of exercise, aging, and disease states. Exercise and Sport Sciences Reviews, 43, 117–124. 25688763 10.1249/JES.0000000000000044 PMC4470710

[phy270639-bib-0025] Hughes, R. L. , Clode, M. , Edwards, R. H. , Goodwin, T. J. , & Jones, N. L. (1968). Effect of inspired O_2_ on cardiopulmonary and metabolic responses to exercise in man. Journal of Applied Physiology, 24, 336–347. 5640720 10.1152/jappl.1968.24.3.336

[phy270639-bib-0026] Keir, D. A. , Iannetta, D. , Mattioni Maturana, F. , Kowalchuk, J. M. , & Murias, J. M. (2022). Identification of non‐invasive exercise thresholds: Methods, strategies, and an online app. Sports Medicine, 52, 237–255. 34694596 10.1007/s40279-021-01581-z

[phy270639-bib-0027] Knight, D. R. , Schaffartzik, W. , Poole, D. C. , Hogan, M. C. , Bebout, D. E. , & Wagner, P. D. (1993). Effects of hyperoxia on maximal leg O_2_ supply and utilization in men. Journal of Applied Physiology, 75, 2586–2594. 8125878 10.1152/jappl.1993.75.6.2586

[phy270639-bib-0028] Manferdelli, G. , Barstow, T. J. , & Millet, G. P. (2023). NIRS‐based muscle oxygenation is suitable for computation of the convective and diffusive components of O_2_ transport at V̇O_2_max. Medicine and Science in Sports and Exercise, 55, 2103–2105. 37343383 10.1249/MSS.0000000000003238

[phy270639-bib-0029] Martin‐Rincon, M. , Gelabert‐Rebato, M. , Perez‐Valera, M. , Galvan‐Alvarez, V. , Morales‐Alamo, D. , Dorado, C. , Boushel, R. , Hallen, J. , & Calbet, J. A. L. (2021). Functional reserve and sex differences during exercise to exhaustion revealed by post‐exercise ischaemia and repeated supramaximal exercise. The Journal of Physiology, 599, 3853–3878. 34159610 10.1113/JP281293

[phy270639-bib-0030] Millet, G. P. , Burtscher, J. , Bourdillon, N. , Manferdelli, G. , Burtscher, M. , & Sandbakk, Ø. (2023). The V˙O2max legacy of hill and Lupton (1923)‐100 years on. International Journal of Sports Physiology and Performance, 18, 1362–1365. 37770066 10.1123/ijspp.2023-0229

[phy270639-bib-0031] Mollard, P. , Bourdillon, N. , Letournel, M. , Herman, H. , Gibert, S. , Pichon, A. , Woorons, X. , & Richalet, J. P. (2010). Validity of arterialized earlobe blood gases at rest and exercise in normoxia and hypoxia. Respiratory Physiology & Neurobiology, 172, 179–183. 20493971 10.1016/j.resp.2010.05.017

[phy270639-bib-0032] Murias, J. M. , Spencer, M. D. , Keir, D. A. , & Paterson, D. H. (2013). Systemic and vastus lateralis muscle blood flow and O2 extraction during ramp incremental cycle exercise. American Journal of Physiology. Regulatory, Integrative and Comparative Physiology, 304, R720–R725. 23515617 10.1152/ajpregu.00016.2013 PMC3652075

[phy270639-bib-0033] Pedersen, P. K. , Kiens, B. , & Saltin, B. (1999). Hyperoxia does not increase peak muscle oxygen uptake in small muscle group exercise. Acta Physiologica Scandinavica, 166, 309–318. 10468668 10.1046/j.1365-201x.1999.00575.x

[phy270639-bib-0034] Peltonen, J. E. , Tikkanen, H. O. , Rusko, H. K. , Peltonen, J. E. , Tikkanen, H. O. , & Rusko, H. K. (2001). Cardiorespiratory responses to exercise in acute hypoxia, hyperoxia and normoxia. European Journal of Applied Physiology, 85(1), 82–88. 11513325 10.1007/s004210100411

[phy270639-bib-0035] Poole, D. C. , Copp, S. W. , Ferguson, S. K. , & Musch, T. I. (2013). Skeletal muscle capillary function: contemporary observations and novel hypotheses. Experimental Physiology, 98, 1645–1658. 23995101 10.1113/expphysiol.2013.073874 PMC4251469

[phy270639-bib-0036] Poole, D. C. , & Musch, T. I. (2008). Solving the Fick principle using whole body measurements does not discriminate “central” and “peripheral” adaptations to training. European Journal of Applied Physiology, 103, 117–119. 18188582 10.1007/s00421-007-0668-4

[phy270639-bib-0037] Porcelli, S. , Pilotto, A. M. , & Rossiter, H. B. (2023). NIRS‐based muscle oxygenation is not suitable to compute convective and diffusive components of O_2_ transport at V̇O_2_max. Medicine and Science in Sports and Exercise, 55, 2106–2109. 37343384 10.1249/MSS.0000000000003239 PMC10592547

[phy270639-bib-0038] Richard, R. , Lonsdorfer‐Wolf, E. , Charloux, A. , Doutreleau, S. , Buchheit, M. , Oswald‐Mammosser, M. , Lampert, E. , Mettauer, B. , Geny, B. , & Lonsdorfer, J. (2001). Non‐invasive cardiac output evaluation during a maximal progressive exercise test, using a new impedance cardiograph device. European Journal of Applied Physiology, 85, 202–207. 11560071 10.1007/s004210100458

[phy270639-bib-0039] Richardson, R. S. , Grassi, B. , Gavin, T. P. , Haseler, L. J. , Tagore, K. , Roca, J. , & Wagner, P. D. (1999). Evidence of O_2_ supply‐dependent VO_2_ max in the exercise‐trained human quadriceps. Journal of Applied Physiology (1985), 86, 1048–1053. 10.1152/jappl.1999.86.3.1048 10066722

[phy270639-bib-0040] Richardson, R. S. , Knight, D. R. , Poole, D. C. , Kurdak, S. S. , Hogan, M. C. , Grassi, B. , & Wagner, P. D. (1995). Determinants of maximal exercise VO_2_ during single leg knee‐extensor exercise in humans. American Journal of Physiology, 268, H1453–H1461. 7733346 10.1152/ajpheart.1995.268.4.H1453

[phy270639-bib-0041] Richardson, R. S. , Leigh, J. S. , Wagner, P. D. , & Noyszewski, E. A. (1999). Cellular PO_2_ as a determinant of maximal mitochondrial O_2_ consumption in trained human skeletal muscle. Journal of Applied Physiology (1985), 87, 325–331. 10.1152/jappl.1999.87.1.325 10409591

[phy270639-bib-0042] Rissanen, A.‐P. E. , Mikkola, T. , Gagnon, D. D. , Lehtonen, E. , Lukkarinen, S. , & Peltonen, J. E. (2024). Wagner diagram for modeling O_2_ pathway—calculation and graphical display by the Helsinki O2 pathway tool. Physiological Measurement, 45, 055028. 10.1088/1361-6579/ad4c36 38749432

[phy270639-bib-0043] Roca, J. , Agusti, A. G. , Alonso, A. , Poole, D. C. , Viegas, C. , Barbera, J. A. , Rodriguez‐Roisin, R. , Ferrer, A. , & Wagner, P. D. (1992). Effects of training on muscle O2 transport at VO_2_max. Journal of Applied Physiology (1985), 73, 1067–1076. 10.1152/jappl.1992.73.3.1067 1400019

[phy270639-bib-0044] Roca, J. , Hogan, M. C. , Story, D. , Bebout, D. E. , Haab, P. , Gonzalez, R. , Ueno, O. , & Wagner, P. D. (1989). Evidence for tissue diffusion limitation of VO_2max_ in normal humans. Journal of Applied Physiology (1985), 67, 291–299. 10.1152/jappl.1989.67.1.291 2759955

[phy270639-bib-0045] Rosales, A. M. , Shute, R. J. , Hailes, W. S. , Collins, C. W. , Ruby, B. C. , & Slivka, D. R. (2022). Independent effects of acute normobaric hypoxia and hypobaric hypoxia on human physiology. Scientific Reports, 12, 19570. 36379983 10.1038/s41598-022-23698-5 PMC9666440

[phy270639-bib-0046] Rowell, L. B. , Saltin, B. , Kiens, B. , & Christensen, N. J. (1986). Is peak quadriceps blood flow in humans even higher during exercise with hypoxemia? The American Journal of Physiology, 251, H1038–H1044. 3777192 10.1152/ajpheart.1986.251.5.H1038

[phy270639-bib-0047] Siebenmann, C. , Rasmussen, P. , Sørensen, H. , Zaar, M. , Hvidtfeldt, M. , Pichon, A. , Secher, N. H. , & Lundby, C. (2015). Cardiac output during exercise: a comparison of four methods. Scandinavian Journal of Medicine & Science in Sports, 25, e20–e27. 24646113 10.1111/sms.12201

[phy270639-bib-0048] Skattebo, Ø. , Calbet, J. A. L. , Rud, B. , Capelli, C. , & Hallén, J. (2020). Contribution of oxygen extraction fraction to maximal oxygen uptake in healthy young men. Acta Physiologica (Oxford), 230, e13486. 10.1111/apha.13486 PMC7540168 32365270

[phy270639-bib-0049] Sperlich, B. , Zinner, C. , Hauser, A. , Holmberg, H.‐C. , Wegrzyk, J. , Sperlich, B. , Zinner, C. , Hauser, A. , Holmberg, H.‐C. , & Wegrzyk, J. (2016). The impact of hyperoxia on human performance and recovery. Sports Medicine, 47(3), 429–438. 10.1007/s40279-016-0590-1 27475952

[phy270639-bib-0050] Stenberg, J. , Ekblom, B. , & Messin, R. (1966). Hemodynamic response to work AT simulated altitude 4,000 m. Journal of Applied Physiology, 21, 1589–1594. 5923231 10.1152/jappl.1966.21.5.1589

[phy270639-bib-0051] Sun, Y. I. , Ferguson, B. S. , Rogatzki, M. J. , McDonald, J. R. , & Gladden, L. B. (2016). Muscle near‐infrared spectroscopy signals versus venous blood hemoglobin oxygen saturation in skeletal muscle. Medicine and Science in Sports and Exercise, 48, 2013–2020. 27635772 10.1249/MSS.0000000000001001

[phy270639-bib-0052] Ulrich, S. , Hasler, E. , Müller‐Mottet, S. , Keusch, S. , Furian, M. , Latshang, T. , Schneider, S. , Saxer, S. , & Bloch, K. (2017). Mechanisms of improved exercise performance under hyperoxia. Respiration, 93, 90–98. 28068656 10.1159/000453620

[phy270639-bib-0053] Wagner, P. D. (1991). Central and peripheral aspects of oxygen transport and adaptations with exercise. Sports Medicine, 11, 133–142. 2047621 10.2165/00007256-199111030-00001

[phy270639-bib-0054] Wagner, P. D. (1992). Gas exchange and peripheral diffusion limitation. Medicine and Science in Sports and Exercise, 24, 54–58. 1548996

[phy270639-bib-0055] Wagner, P. D. (2011). Modeling O_2_ transport as an integrated system limiting VO_2_MAX. Computer Methods and Programs in Biomedicine, 101, 109–114. 20483502 10.1016/j.cmpb.2010.03.013 PMC2939967

[phy270639-bib-0056] Wagner, P. D. (2023). Determinants of maximal oxygen consumption. Journal of Muscle Research and Cell Motility, 44, 73–88. 36434438 10.1007/s10974-022-09636-y

[phy270639-bib-0057] Wehrlin, J. P. , & Hallén, J. (2006). Linear decrease in VO_2_max and performance with increasing altitude in endurance athletes. European Journal of Applied Physiology, 96, 404–412. 16311764 10.1007/s00421-005-0081-9

[phy270639-bib-0058] Zavorsky, G. S. , Cao, J. , Mayo, N. E. , Gabbay, R. , & Murias, J. M. (2007). Arterial versus capillary blood gases: a meta‐analysis. Respiratory Physiology & Neurobiology, 155, 268–279. 16919507 10.1016/j.resp.2006.07.002

